# Toward Detecting Infection Incidence in People With Type 1 Diabetes Using Self-Recorded Data (Part 1): A Novel Framework for a Personalized Digital Infectious Disease Detection System

**DOI:** 10.2196/18911

**Published:** 2020-08-12

**Authors:** Ashenafi Zebene Woldaregay, Ilkka Kalervo Launonen, Eirik Årsand, David Albers, Anna Holubová, Gunnar Hartvigsen

**Affiliations:** 1 Department of Computer Science University of Tromsø – The Arctic University of Norway Tromsø Norway; 2 Department of Clinical Research University Hospital of North Norway Tromsø Norway; 3 Department of Pediatrics, Informatics and Data Science University of Colorado Aurora, CO United States; 4 Department of Biomedical Informatics Columbia University New York, NY United States; 5 Department of ICT in Medicine Faculty of Biomedical Engineering Czech Technical University Prague Czech Republic; 6 Spin-off Company and Research Results Commercialization Center of the First Faculty of Medicine Charles University Prague Czech Republic

**Keywords:** type 1 diabetes, self-recorded health data, infection incidence, decision making, infectious disease outbreaks, public health surveillance

## Abstract

**Background:**

Type 1 diabetes is a chronic condition of blood glucose metabolic disorder caused by a lack of insulin secretion from pancreas cells. In people with type 1 diabetes, hyperglycemia often occurs upon infection incidences. Despite the fact that patients increasingly gather data about themselves, there are no solid findings that uncover the effect of infection incidences on key parameters of blood glucose dynamics to support the effort toward developing a digital infectious disease detection system.

**Objective:**

The study aims to retrospectively analyze the effect of infection incidence and pinpoint optimal parameters that can effectively be used as input variables for developing an infection detection algorithm and to provide a general framework regarding how a digital infectious disease detection system can be designed and developed using self-recorded data from people with type 1 diabetes as a secondary source of information.

**Methods:**

We retrospectively analyzed high precision self-recorded data of 10 patient-years captured within the longitudinal records of three people with type 1 diabetes. Obtaining such a rich and large data set from a large number of participants is extremely expensive and difficult to acquire, if not impossible. The data set incorporates blood glucose, insulin, carbohydrate, and self-reported events of infections. We investigated the temporal evolution and probability distribution of the key blood glucose parameters within a specified timeframe (weekly, daily, and hourly).

**Results:**

Our analysis demonstrated that upon infection incidence, there is a dramatic shift in the operating point of the individual blood glucose dynamics in all the timeframes (weekly, daily, and hourly), which clearly violates the usual norm of blood glucose dynamics. During regular or normal situations, higher insulin and reduced carbohydrate intake usually results in lower blood glucose levels. However, in all infection cases as opposed to the regular or normal days, blood glucose levels were elevated for a prolonged period despite higher insulin and reduced carbohydrates intake. For instance, compared with the preinfection and postinfection weeks, on average, blood glucose levels were elevated by 6.1% and 16%, insulin (bolus) was increased by 42% and 39.3%, and carbohydrate consumption was reduced by 19% and 28.1%, respectively.

**Conclusions:**

We presented the effect of infection incidence on key parameters of blood glucose dynamics along with the necessary framework to exploit the information for realizing a digital infectious disease detection system. The results demonstrated that compared with regular or normal days, infection incidence substantially alters the norm of blood glucose dynamics, which are quite significant changes that could possibly be detected through personalized modeling, for example, prediction models and anomaly detection algorithms. Generally, we foresee that these findings can benefit the efforts toward building next generation digital infectious disease detection systems and provoke further thoughts in this challenging field.

## Introduction

The incidence of infectious disease outbreaks can create panic in society and is a threat to local and global health security. Such outbreaks require immediate detection and appropriate response during the initial phase of the incidence to reduce fatality and save lives [1]. The timeliness of outbreak detection defines the success of the appropriate response by the concerned bodies. The state-of-the-art syndromic surveillance systems have been improved compared with the traditional surveillance system, which is generally passive and dependent on laboratory confirmation [2]. Syndromic surveillance makes use of features that come before diagnosis, including different activities triggered by the onset of symptoms, such as Google search, Twitter, school and work absenteeism, pharmacy drug sells, and other sources as a signal of change in individual and population health [2]. These signals are mainly acquired from the secondary source of information, typically built for other purposes. However, to keep up the pace with the rapidly changing social and biological dynamics, novel outbreak detection mechanisms are highly sought [2].

The advancement and omnipresence of smartphones, Internet of Things (IoT) devices, wearables, and sensors have enabled individuals to easily self-record health-related events often for self-tracking or self-managing their disease [3,4]. The recent movement known as quantified self and lifelogging is the result of such technological advancement, where people collect various kinds of health-related events and data for personal informatics purposes, that is, self-surveillance and self-management [5-8]. To this end, people with diabetes are not an exception, where they self-record detailed information as part of their self-management, including blood glucose levels, diet and insulin intake, physical activity, medication, and other information [4,9,10]. Consequently, a huge amount of self-recorded, personal health-related data is generated each day that have great potential to be used as a secondary source of information for other purposes such as digital epidemiology [11,12]. According to recent reports, personal health data or self-collected health-related data have provided an enormous opportunity to enhance the possibility of detecting infection incidence during the presymptomatic stage (improved sensitivity and timeliness), specifically during the incubation period, where most of the existing systems neglect from their process [13].

Type 1 diabetes is a chronic condition of blood glucose metabolic disorder caused by lack of insulin secretion from pancreas cells [14]. These patient groups are recommended to maintain their blood glucose levels within a specified range through self-management practice [14,15]. Blood glucose levels are controlled by balancing insulin and meal intake along with other contexts such as physical activity, medications, and others. Blood glucose dynamics are affected by various factors that can be categorized as common, individual, and unpredictable factors [16]. These factors could be further categorized as patient-controllable and patient-uncontrollable parameters [17]. Patient-controllable parameters incorporate factors on which the patient has direct control and can roughly understand their immediate effect on blood glucose dynamics. However, patient-uncontrollable parameters include factors in which the patient does not have direct control and faces a challenge to understand their immediate effect on blood glucose levels. From the patient perspective, usually patient-controllable parameters induce reasonable deviations on blood glucose levels; however, patient-uncontrollable parameters induce unreasonable blood glucose deviations and usually differ from the usual norm of blood glucose dynamics [18]. The total number of people living with diabetes is increasing worldwide. According to recent reports [14], there were 415 million people between the ages of 20 and 79 years in 2015, and this value is projected to increase by 54% in 2040. From this figure, 5% are believed to have type 1 diabetes. In these patient groups, infection incidence often results in complications and difficulties in controlling blood glucose levels within the recommended range [19-21]. As a result, early detection of infection incidence among these patient groups could provide a way to assist the individual and at the same time can be used to realize a digital infectious disease detection system.

Currently, with the advancement of technology, the need to have a system that is able to detect infection incidence at the presymptomatic stage is highly sought [13]. In this regard, there are some previous investigations that have showcased the use of self-recorded data from people with diabetes as surveillance events (indicators) by uncovering the effect of infection incidence on blood glucose levels and glycemic control in real-life settings [18,22-36]. These studies reported the presence of prolonged hyperglycemia episodes as a result of infection incidence, thereby revealing the potential of self-recorded data as a secondary source of information for realizing a digital infectious disease detection system. For instance, Botsis et al [22] conducted a proof-of-concept study based on daily glycemic control data of 248 people with type 2 diabetes and concluded that blood glucose levels, insulin dosage, diet (carbohydrate consumption), physical activity, and other physiological parameters could be used as potential event indicators of infection incidence but calls for further investigations. Furthermore, Botsis et al [18] also reported elevated glycated hemoglobin (HbA_1c_) levels after infections regardless of tight blood glucose control, which only settled down to normal levels after the patient recovered. Moreover, other studies conducted in hospital settings also reported similar results in this direction [37,38]. Despite reporting the potential of using self-recorded data as a surveillance event indicator, none of these studies demonstrated the extent to which each parameter is affected at an individual level as a result of infection incidence. Therefore, the purpose of this study was to retrospectively analyze the effect of infection incidence at an individual level and pinpoint optimal parameters that can effectively be used as input variables for developing an infection detection algorithm, thereby illustrating how these patient groups can assist in detecting infectious disease outbreaks. Moreover, this study provides a general framework regarding how a digital infectious disease detection system can be designed using self-recorded data from people with type 1 diabetes as a secondary source of information. Furthermore, this sheds light on the possibility of assisting the individual during such an incident. To this end, we analyzed temporal trends and probability distributions of different diabetes profile parameters (ie, blood glucose, insulin, carbohydrate, and others) to uncover the effect of infection incidence on the blood glucose dynamics, thereby identifying parameters that can effectively be used as potential events (indicators) of infection incidence. In addition, a framework is presented depicting the necessary structure to properly exploit self-recorded data from these patient groups to realize a real-time digital infectious disease detection system. This paper is structured as follows: the Methods section describes the materials and methods used to analyze the data sets. The Results section presents the results depicting the effect of acute infection incidence in comparison with regular or normal situations. The Discussion section presents the overall findings and proposes a framework for designing and developing a real-time digital infectious disease detection system using self-recorded data from these patient groups. The final section of *Discussion* presents our concluding remarks.

## Methods

### Materials

High precision self-recorded data of 10 patient years collected from 3 real subjects (2 males and 1 female) with type 1 diabetes were used. The patients were free from any other chronic or other form of disease, except the self-reported acute infection incidence throughout the entire data collection period. The data sets consisted of blood glucose measurements (self-monitoring of blood glucose [SMBG] and continuous glucose monitoring [CGM]), injected insulin (basal and bolus), diet (carbohydrate in grams), and self-reported events of acute infection. The patients used different diabetes self-management technologies throughout the data collection period to gather these data sets including the Diabetes Diary app (Norwegian Centre for E-health Research) [39], the Spike app [40], the xDrip with app, Dexcom CGM, insulin pens, and insulin pumps, as shown in Table 1. The data sets consist of both normal years, without any significant acute infection incidence, and years with at least one or more acute infection incidence. The normal (without infection) patient years were used as a baseline to compare the effect of all patient-controllable parameters and patient-uncontrollable parameters against the self-reported incidence of acute infection. The self-reported incidences of acute infections were a case of influenza (flu) and mild and light common cold without fever. All the experiments and analyses were conducted using MATLAB version 2018a (Mathworks).

**Table 1:** Equipment used in diabetes self-management.

Patients	Self-management		
	BG^a^	Insulin administration	Diet
Subject 1	SMBG^b^—finger pricks recorded in the Diabetes Diary mobile app and Dexcom CGM^c^	Insulin pen (multiple bolus and one-time basal in the morning) recorded in the Diabetes Diary mobile app	Carbohydrate in grams recorded in the Diabetes Diary mobile app
Subject 2	SMBG—finger pricks recorded in the Spike mobile app and Dexcom G4 CGM	Insulin pen (multiple bolus [Humalog] and one-time basal [Toujeo] before bed) recorded in the Spike mobile app	Carbohydrate in grams recorded in the Spike mobile app
Subject 3	Enlite (Medtronic) CGM and Dexcom G4 CGM	Medtronic MinMed G640 insulin pump (basal rates profile [Fiasp] and multiple bolus [Fiasp])	Carbohydrate in grams recorded in pump information

^a^BG: blood glucose.

^b^SMBG: self-monitoring of blood glucose.

^c^CGM: continuous glucose monitoring.

#### Patient Characteristics

The participants were highly motivated individuals with type 1 diabetes who had advanced knowledge and understanding of several diabetes-related technologies. Hence, the self-recorded data can be regarded as highly precise and accurate. All the participants had advanced knowledge of carbohydrate counting, which can be considered as level 3 (advanced) [41]. The long-term average HbA_1c_ and characteristics of the participants are given in Table 2.

**Table 2:** Participants characteristics.

Variables		Values	
**Gender, n**			
	Male		2
	Female		1
Age (years), mean (SD)		34 (13.2)	
**Body weight (kg)**			
	Subject 1		83
	Subject 2		77
	Subject 3		70
**HbA_1c_^a^(%)**			
	Subject 1		6.0
	Subject 2		7.3
	Subject 3		6.2
Carbohydrate counting		Level 3 (advanced)	

^a^HbA_1c_: glycated hemoglobin.

#### Data Collection and Ethics

The study protocol has been submitted to the Norwegian Regional Committees for Medical Health Research Ethics Northern Norway (REK) for evaluation and was found exempted from regional ethics review because it resides outside of the scope of medical research (reference number: 108435). Written consent was obtained and the participants donated the data sets. All data from the participants were anonymized.

### Approaches

We retrospectively assessed and analyzed the diabetes profile (blood glucose, insulin, carbohydrate, and insulin-to-carbohydrate ratio) to uncover the nature, size, and shape of the infection-induced shift in the operating region of the blood glucose dynamics. A data size of 10 patient years incorporating blood glucose levels (SMBG and CGM), insulin (bolus and basal), diet (carbohydrate in grams), and self-reported events of acute infection was used. The analysis was performed based on specified timeframes (weekly, daily, and hourly) to reveal the effect of acute infection development on blood glucose dynamics. The data set incorporates 5 normal patient years without any infection incidence and 5 patient years each with at least one case of self-reported incidence of acute infection. Normal patient years were used as a baseline for comparison purposes. We analyzed the temporal evolution and probability distribution of blood glucose levels, injected insulin, carbohydrate intake (grams), and insulin-to-carbohydrate ratio within the stated timeframe. For the daily and hourly timeframes, a moving-average filter and nonparametric density estimation techniques, the kernel density estimator, were used to analyze the trend and data distribution before, during, and after the infection incidence. A moving-average filter with a window size of 2 days was employed to remove fast timescale features through smoothing. The window size includes *N*−1 observations from the previous data points and the current data point, where *N* is the window size. Generally, the window size of a moving-average filter is determined based on complementary issues of better smoothing and the cost of significant delay (shift) incurred [42,43]. A small window size often generates less delay (shift) but at the cost of more short-term features and having a larger window size will smoothen the data in a better manner but at the cost of significant delay in the timeliness of detecting the infection incidence. Therefore, the window size was determined based on these complementary issues, and more importance was given to minimize the inherent delay (shift) incurred due to the window size. To this end, window sizes of 1, 2, 3, and 4 days were applied and tested to choose the optimal size of the window, and as a result, a window size of 2 days was found to be satisfactory. The preinfection, infection, and postinfection week analyses were carried out on the raw data set based on the week’s daily average and SD of blood glucose levels and daily sum and SD of insulin and carbohydrate. A statistical boxplot was used to depict the comparison during preinfection, infection, and postinfection weeks.

#### Data Resampling, Imputation, and Preprocessing

The features of the self-collected data from individuals with type 1 diabetes are shown in Table 3. The raw data were resampled at a uniform rate by assigning each measurement into the nearest time-bin based on its time stamp. Generally, whenever there is more than one measurement within each time-bin, the measurements are combined into a single measurement by either summing or averaging the elements. For blood glucose levels (both CGM and SMBG), the measurements were averaged into their respective sampling time-bins. However, regarding carbohydrate consumption and insulin injections, the sum of the elements in their respective sampling time-bin was computed, as shown in Table 4. In each time-bin, the effect of total insulin and total carbohydrate on the average blood glucose level was considered. The resampled data were further preprocessed using a moving-average filter with a 2-day (48-hour) window size to capture only the important patterns—long-term variation, while filtering and smoothing local and short-term variations. Moreover, for narrower time-bin resampling, for example, an hour, there are more frequent zeros of measurement, especially for carbohydrate and insulin measurements, which poses a significant challenge to compute the insulin-to-carbohydrate ratio as the ratio goes to infinity given that the carbohydrate amount is zero. Therefore, in such cases of a narrower time-bin, the ratio was computed only after computing the moving-average value of insulin and carbohydrate based on a window size of 48 hours. Regarding the missing blood glucose values during the hourly computations, a cubic spline interpolation was used to estimate the missing values.

**Table 3:** Self-collected user data.

Variable names	Subject’s record variables	
	Description	Units
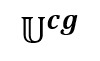	Continuous glucose reading	mg/dL
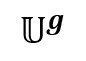	Self-management blood glucose reading	mg/dL
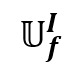	Injected insulin (bolus)	Units
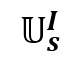	Injected insulin (basal)	Units
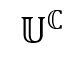	Ingested carbohydrate	Grams

**Table 4:** Data preprocessing.

Variable name	Preprocessed variables	
	Description	Units
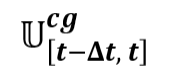	Average continuous glucose reading	mg/dL
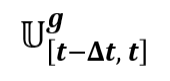	Average self-management blood glucose reading	mg/dL
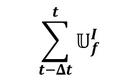	Sum injected insulin (bolus)	Units
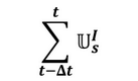	Sum injected insulin (basal)	Units
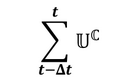	Sum ingested carbohydrate	Grams
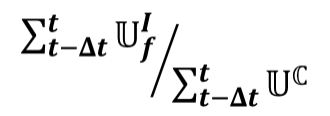	Ratio of insulin (bolus) to carbohydrate	Units/grams
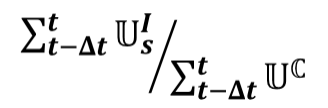	Ratio of insulin (basal) to carbohydrate	Units/grams

#### Kernel Density Estimation

Nonparametric density estimation is an alternative to the parametric approach, which involves specifying a model using a number of parameters that can be estimated through the likelihood principle [44,45]. In this study, we used kernel density estimation techniques [46-48] to estimate the probability distribution of the diabetes profile key parameters to uncover the deviation incurred by the acute infection incidence. In this regard, both univariate and bivariate kernel density estimators are used to assess and analyze the insulin-to-carbohydrate ratio (univariate) and blood glucose levels along with the insulin-to-carbohydrate ratio (bivariate), respectively. An adaptive kernel density estimator with a Gaussian kernel was used in both cases***.*** For the univariate kernel density estimator [49], bandwidth selection is based on the suggestion from Botev et al [44], which is a data-driven and plug-in bandwidth selector that does not use normal reference rules. For the bivariate estimator, a rule-of-thumb bandwidth selection suggested by Bowman et al [50,51] was used to determine the appropriate bandwidth [52]. These computations are carried out based on the procedures given in Textboxes 1 and 2.

**One-dimensional adaptive kernel density estimation.**

Approach: one-dimensional adaptive kernel density estimation

- Given: time series data sets of the insulin-to-carbohydrate ratio X ε D and an adaptive kernel density estimator *M – one – dimensional*
- Remove the reported days of infection from the time series data sets D and form a new data set *X* ε Q
- Compute the one dimensional density based on the kernel density estimator *M* using D and Q
- Compare the distribution from *M*

**Two-dimensional adaptive kernel density estimation.**

Approach: two-dimensional adaptive kernel density estimation

- Given: time series data sets of blood glucose level and the insulin-to-carbohydrate ratio X, Y ε D and an adaptive kernel density estimator N – *two - dimensional*
- Remove the reported days of infection from the time series data sets D and form a new data set *X*, *Y* ε Q
- Compute the two-dimensional density based on the kernel density estimator N using D and Q
- Compare the distribution from N

## Results

### Overview

The analysis was conducted based on an hourly, daily, and weekly basis to reveal the deviations incurred due to the infection incidence. A total of 10 patient years were analyzed, and 5 of these years were found to include at least one incidence of acute infection lasting around 1-2 weeks. The proposed approach is designed to smooth out short-duration variations and include the 2 major patient-controllable factors, insulin and diet intake. Normal patient years were used to compare the effect of all patient-controllable parameters and patient-uncontrollable parameters against the self-reported incidence of acute infection. The trend analysis for both the normal patient years and patient years with acute infections using the proposed approach is presented below along with the nonparametric probability distribution. The weekly mean deviations of key diabetes parameters (blood glucose, insulin, and diet) during the preinfection, infection, and postinfection weeks are given in Multimedia Appendix 1.

### Trend Analysis

#### Trend Comparison for Normal Patient Years

**Figure 1:**
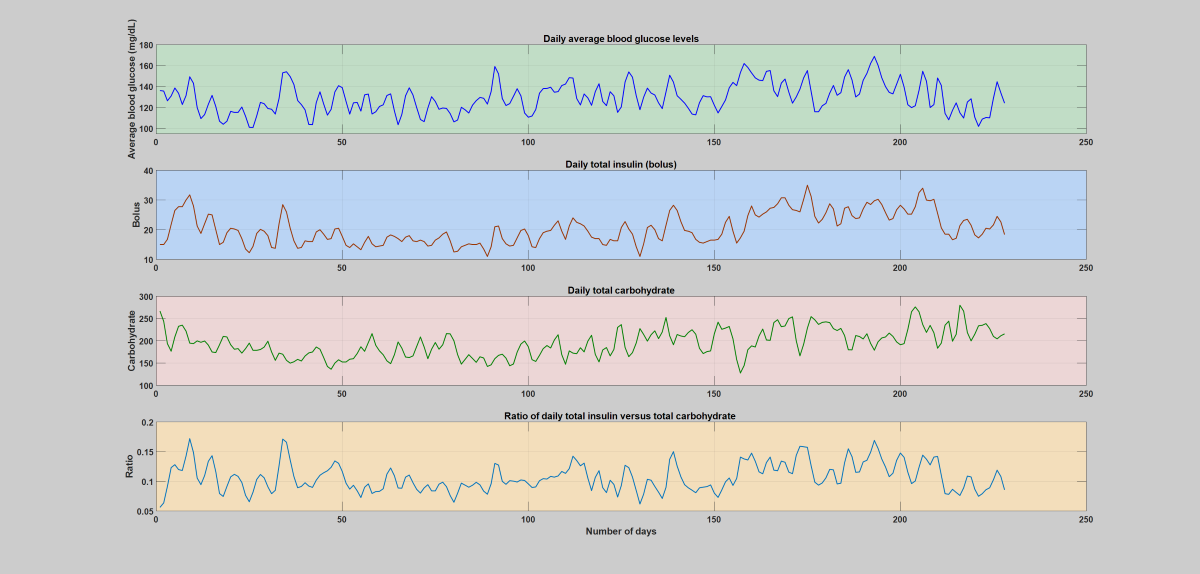
The first patient year, where there is no incidence of acute infections. The figure depicts the daily variation of average blood glucose levels, total insulin (bolus), total carbohydrate, and total insulin-to-total carbohydrate ratio. The operating point of the patient’s insulin-to-carbohydrate ratio through these regular or normal days is between 0.05-0.2.

**Figure 2:**
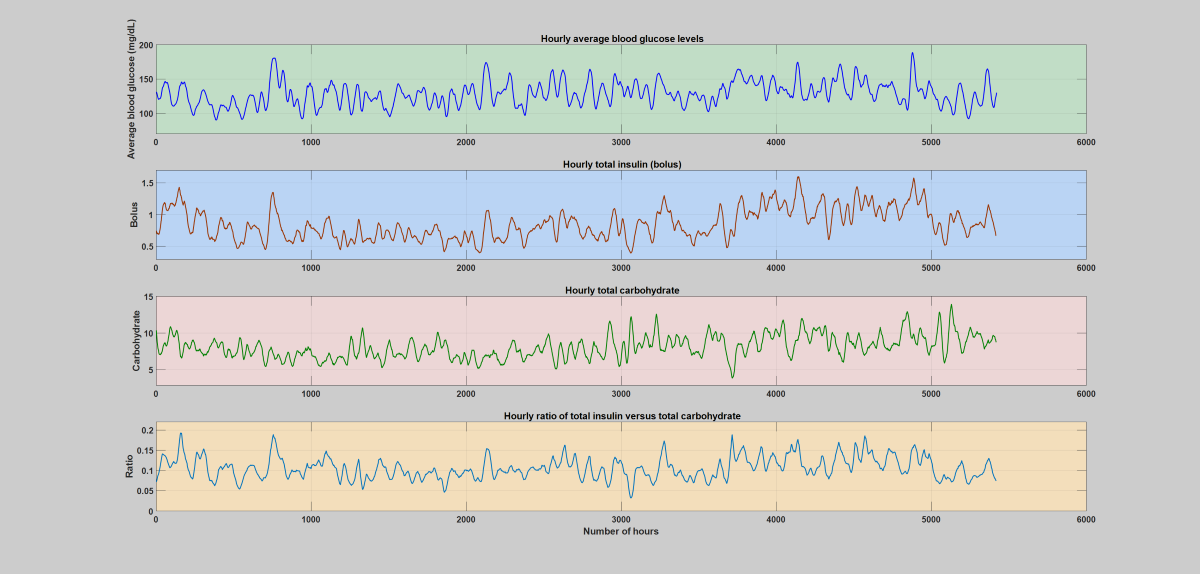
The first patient year, where there is no incidence of acute infections. The figure depicts variation of average blood glucose levels, total insulin (bolus), total carbohydrate, and total insulin-to-carbohydrate ratio during each hours of the day. The operating point of the patient’s insulin-to-carbohydrate ratio through these regular or normal hours is between 0.05-0.2.

During normal years when patients do not have any significant illness or infections (Multimedia Appendix 2), the insulin-to-carbohydrate ratio follows a similar trend in all the subjects, where the insulin-to-carbohydrate ratio lies between 0.05 and 0.2. An elaborate analytical plot of a typical patient year without infection incidence showing the phenomena is depicted in Figures 1 and 2. A detailed analytical plot of the 5 patient years depicting the same phenomena can be found in Multimedia Appendix 2. The insulin-to-carbohydrate ratio conveys interesting information about the usual operating point of the patient, depicting the necessary amount of insulin (bolus) required for every gram of carbohydrate consumed to maintain the blood glucose levels within a healthy range (typically recommended to be between 70 and 180 mg/dL). As can be seen from the yearlong trend analysis of the regular or normal patient years (Multimedia Appendix 2), despite the presence of various factors that are known to disturb blood glucose dynamics, both patient-controllable parameters and patient-uncontrollable parameters except infection incidence, the insulin-to-carbohydrate ratio remains to be relatively stable.

#### Trend Comparison of Patient Years With Acute Infection

The trend analysis of the key diabetes parameters, blood glucose, insulin, and carbohydrate, during acute infection suggests that there is a dramatic shift in the evolution of blood glucose, insulin, and carbohydrate (for detailed information, see Multimedia Appendices 1 and 3). Infection incidence brought about a dramatic increase in blood glucose levels, insulin intake, and reduction in carbohydrate consumption. The detailed analysis and the shift incurred on a weekly, daily, and hourly basis are presented in the following section.

#### Weekly Analysis

**Figure 3:**
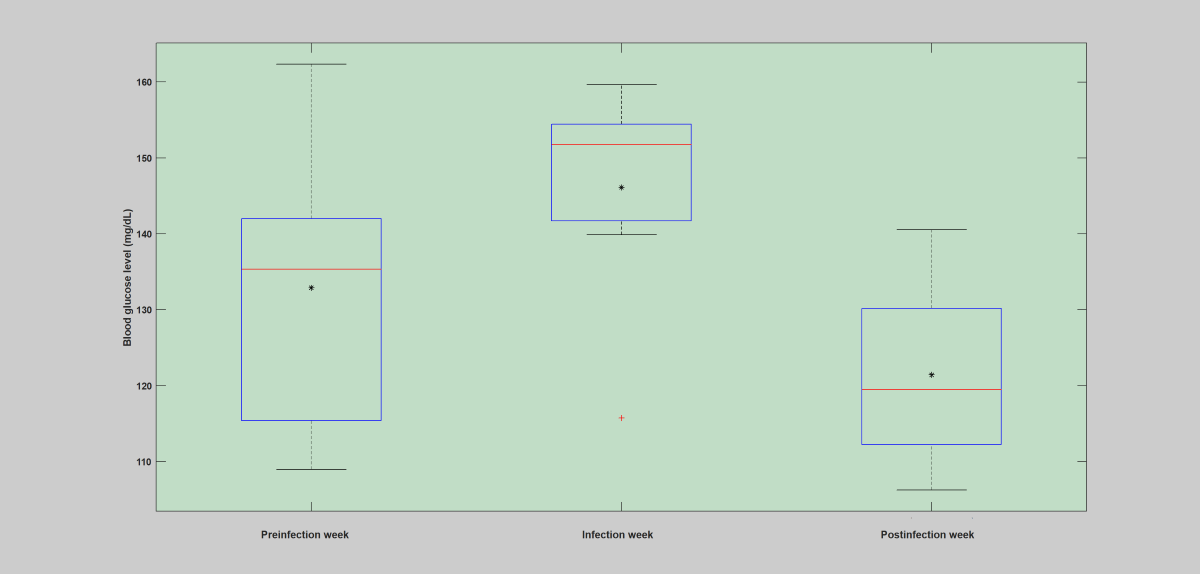
Analysis of blood glucose levels during the preinfection week, infection week, and postinfection week based on the first case of infection (flu). The asterisk shows the mean value, and the red line depicts the median value for the week.

**Figure 4:**
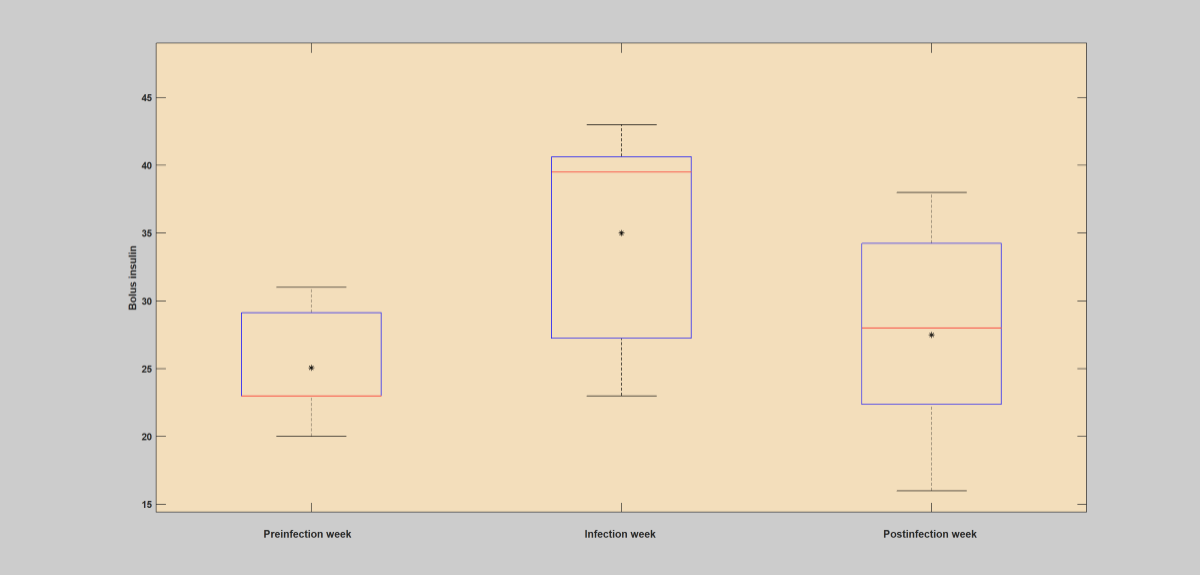
Analysis of total insulin (bolus) intake during preinfection week, infection week, and postinfection week based on the first case of infection (flu). The asterisk shows the mean value, and the red line depicts the median value for the week.

**Figure 5:**
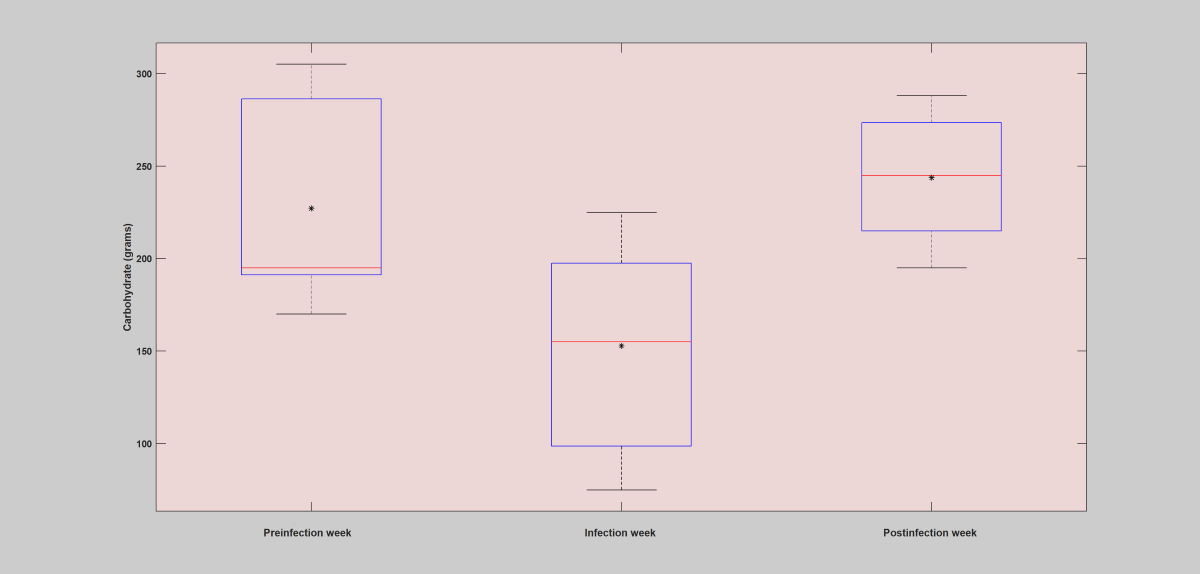
Analysis of total carbohydrate (grams) intake during preinfection week, infection week, and postinfection week based on the first case of infection (flu). The asterisk shows the mean value, and the red line depicts the median value for the week.

The weekly analysis of the patient years was conducted by analyzing the deviation incurred on the key parameters of the blood glucose dynamics during the infection week in comparison with before and after the infection incidence. The raw data were used to estimate the deviations incurred due to infection incidence. The mean and SD of blood glucose levels, total insulin (bolus), and total carbohydrate were computed and used for comparison of the infection-induced deviations. As shown in Figures 3-5 and Table 5, in all the infection cases, the weekly analysis demonstrated that blood glucose levels were elevated despite higher insulin injection and reduced carbohydrate consumption. In all of these cases, it is clear that the incidence of infection has brought unreasonable deviation, with respect to the patient-controllable parameters, in the operation of the overall blood glucose dynamics as compared with the usual norm of the blood glucose dynamics. The presence of elevated blood glucose levels in the infection week, regardless of the high amount of insulin injections and lower carbohydrate consumption, clearly violated the norm of the blood glucose dynamics, where during normal situations the blood glucose levels are expected to drop with high insulin and reduced carbohydrate consumption. The fact that the blood glucose remains elevated during the infection incidence despite higher insulin injections and low carbohydrate consumption is highly associated with the infection phenomenon, which enhances the production of glucose and increased insulin resistance within the body to deliver more energy for the body to fight the pathogens. A more detailed description of the weekly analysis can be found in Multimedia Appendix 1.

**Table 5:** Mean and standard deviation of blood glucose levels, total insulin (bolus), and total carbohydrate during the preinfection week, infection week, and postinfection week.

Parameters		Preinfection week, mean (SD)	Infection week, mean (SD)	Postinfection week, mean (SD)
**The first case of infection (flu)**				
	BG^a^ (mg/dL)	130.74 (16.89)	141.95 (14.37)	119.16 (7.39)
	Total insulin (bolus)	23.39 (4.91)	35.30 (6.11)	21.32 (4.61)
	Carbohydrate (grams)	241.11 (57.27)	178.80 (65.69)	241.18 (37.63)
**The second case of infection (flu)**				
	BG (mg/dL)	143.01 (19.53)	155.36 (21.99)	126.17 (11.70)
	Total insulin (bolus)	28.07 (8.85)	41.07 (9.44)	25.36 (6.93)
	Carbohydrate (grams)	190.14 (43.93)	161.14 (58.43)	214.57 (34.66)
**The third case of infection (flu)**				
	BG (mg/dL)	136.93 (18.58)	144.12 (20.30)	134.18 (11.96)
	Total insulin (bolus)	20.08 (5.44)	31.50 (10.84)	22.83 (3.86)
	Carbohydrate (grams)	178.0 (45.87)	144.83 (37.63)	195.83 (42.59)
**The fourth case of infection (flu)**				
	BG (mg/dL)	157.74 (31.12)	161.34 (19.88)	138.57 (19.83)
	Total insulin (bolus)	24.43 (5.26)	32.14 (7.01)	29.29 (5.22)
	Carbohydrate (grams)	199.06 (53.45)	167.04 (44.94)	226.07 (18.23)
**The fifth case of infection (flu)**				
	BG (mg/dL)	135.21 (14.58)	139.88 (15.54)	122.87 (14.49)
	Insulin (bolus)	32.80 (4.59)	40.37 (8.31)	33.36 (7.94)
	Insulin (basal)	19.20 (1.21)	20.42 (2.06)	18.68 (1.56)
	Total insulin	52.33 (5.14)	61.21 (8.26)	52.46 (8.47)

^a^BG: blood glucose.

### Blood Glucose Levels

In all these infection incidences, the individual blood glucose levels remain elevated for a prolonged period of time despite low carbohydrate consumption and increased insulin injections as compared with the regular or normal days. Blood glucose levels were elevated during the infection week as compared with the preinfection and postinfection weeks.

- During the first case of infection, the overall mean percentage increase in the infection week’s blood glucose levels was 8.57% over the preinfection week and 19.12% over the postinfection week, as shown in Table 5.
- During the second case of infection, the overall mean percentage increase in the infection week’s blood glucose levels was 8.63% over the preinfection week and 23.13% over the postinfection week, as shown in Table 5.
- During the third case of infection, the overall mean percentage increase in the infection week’s blood glucose levels was 7.26% over the preinfection week and 7.41% over the postinfection week, as shown in Table 5.
- During the fourth case of infection, the overall mean percentage increase in the infection week’s blood glucose levels was 2.28% over the preinfection week and 16.43% over the postinfection week, as shown in Table 5.
- During the fifth case of infection, the overall mean percentage increase in the infection week’s blood glucose levels was 3.45% over the preinfection week and 13.84% over the postinfection week, as shown in Table 5.

### Insulin Intake

The comparison of infection week insulin injections with preinfection and postinfection weeks revealed that there was a dramatic increase in the amount of insulin intake during the infection period.

- During the first case of infection, the overall mean percentage increase in the infection week’s insulin (bolus) injection was 50.93% over the preinfection week and 65.59% over the postinfection week, as shown in Table 5.
- During the second case of infection, the overall mean percentage increase in the infection week’s insulin (bolus) injection was 46.31% over the preinfection week and 61.94% over the postinfection week, as shown in Table 5.
- During the third case of infection, the overall mean percentage increase in the infection week’s insulin (bolus) injection was 56.87% over the preinfection week and 37.98% over the postinfection week, as shown in Table 5.
- During the fourth case of infection, the overall mean percentage increase in the infection week’s insulin (bolus) injection was 31.56% over the preinfection week and 9.7% over the postinfection week, as shown in Table 5.
- During the fifth case of infection, the overall mean percentage increase in the infection week’s insulin (bolus) injection was 23.08% over the preinfection week and 21.01% over the postinfection week, as shown in Table 5.

### Carbohydrate Consumption

Comparison of the amount of carbohydrate consumption during the infection week with the preinfection and postinfection weeks revealed that there was a significant reduction during the infection period.

- During the first case of infection, the overall mean percentage reduction in the infection week’s carbohydrate consumption was 25.84% below the preinfection week and 25.87% below the postinfection week, as shown in Table 5.
- During the second case of infection, the overall mean percentage reduction in the infection week’s carbohydrate consumption was 15.25% below the preinfection week and 24.90% below the postinfection week, as shown in Table 5.
- During the third case of infection, the overall mean percentage increase in the infection week’s carbohydrate consumption was 18.63% below the preinfection week and 26.04% below the postinfection week, as shown in Table 5.
- During the fourth case of infection, the overall mean percentage increase in the infection week’s carbohydrate consumption was 16.09% below the preinfection week and 35.34% below the postinfection week, as shown in Table 5.

### Insulin-to-Carbohydrate Ratio

The insulin-to-carbohydrate ratio defines the amount of insulin a patient needs to take for every gram of carbohydrate consumed. The value of the insulin-to-carbohydrate ratio usually lies between 0.05 and 0.2 on normal occasions. However, it has dramatically increased upon the incidence of infection.

- During the first case of infection, the overall mean percentage increase in the infection week’s insulin-to-carbohydrate ratio was around 125.84% above the normal operating point of the patient, as shown in Table 5.
- During the second case of infection, the overall mean percentage increase in the infection week’s insulin-to-carbohydrate ratio was approximately 144.43% above the normal operating point of the patient, as shown in Table 5.
- During the first case of infection, the overall mean percentage increase in the infection week’s insulin-to-carbohydrate ratio was around 93.75% above the normal operating point of the patient, as shown in Table 5.
- During the fourth case of infection, the overall mean percentage increase in the infection week’s insulin-to-carbohydrate ratio was approximately 70.84% above the normal operating point of the patient, as shown in Table 5.

#### Daily and Hourly Analysis

**Figure 6:**
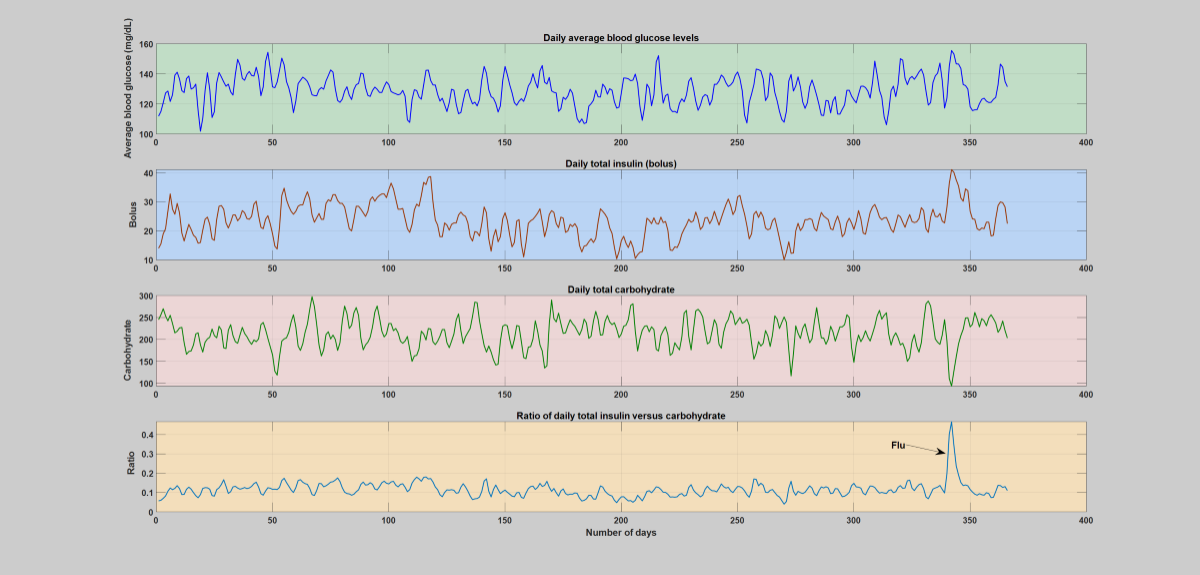
Daily analysis of the first infection case (flu). The figure depicts variation of average blood glucose levels, total insulin (bolus), total carbohydrate, and total insulin-to-total carbohydrate ratio. The operating point of the patient’s insulin-to-carbohydrate ratio had dramatically shifted and raised above the regular or normal days and reach a top around 0.5 upon midinfection week.

**Figure 7:**
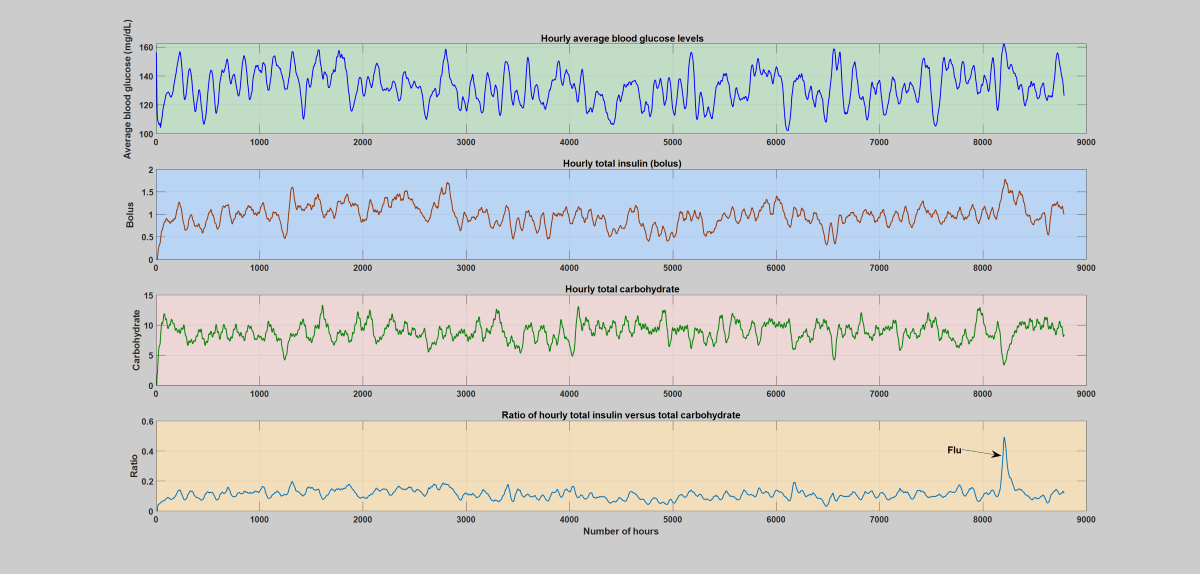
Hourly analysis of the first infection case (flu). The figure depicts variation of average blood glucose levels, total insulin (bolus), total carbohydrate, and total insulin-to-total carbohydrate ratio. The operating point of the patient’s insulin-to-carbohydrate ratio had dramatically shifted and raised above the regular or normal days and reach a top around 0.5 upon midinfection week.

**Figure 8:**
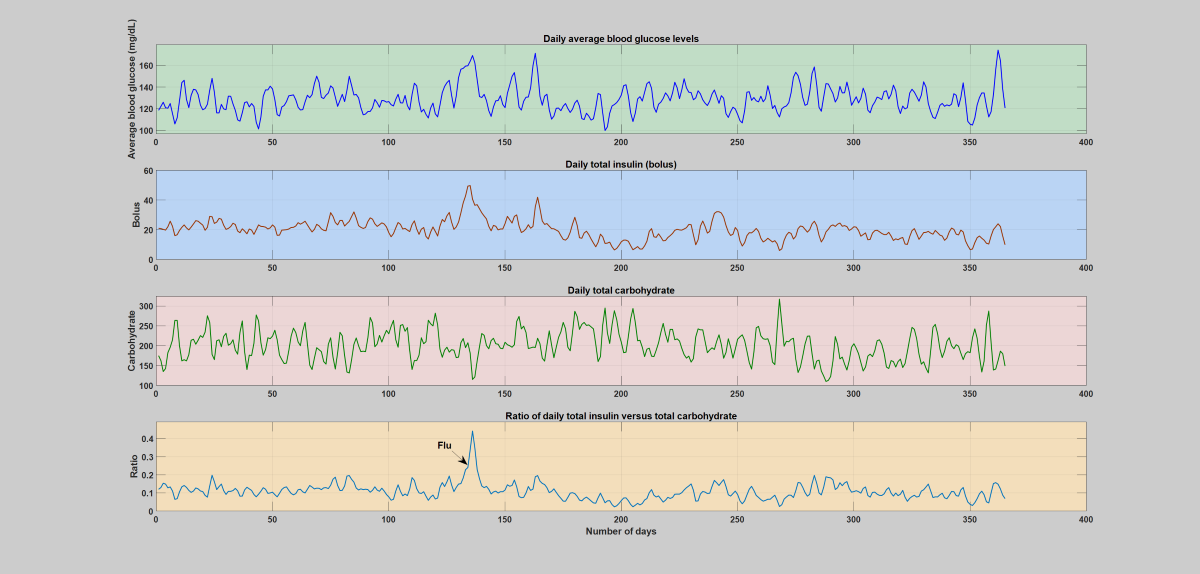
Daily analysis of the second infection case (flu). The figure depicts variation of average blood glucose levels, total insulin (bolus), total carbohydrate, and total insulin-to-total carbohydrate ratio. The operating point of the patient’s insulin-to-carbohydrate ratio had dramatically shifted and raised above the regular or normal days and reach a top around 0.45 upon midinfection week.

**Figure 9:**
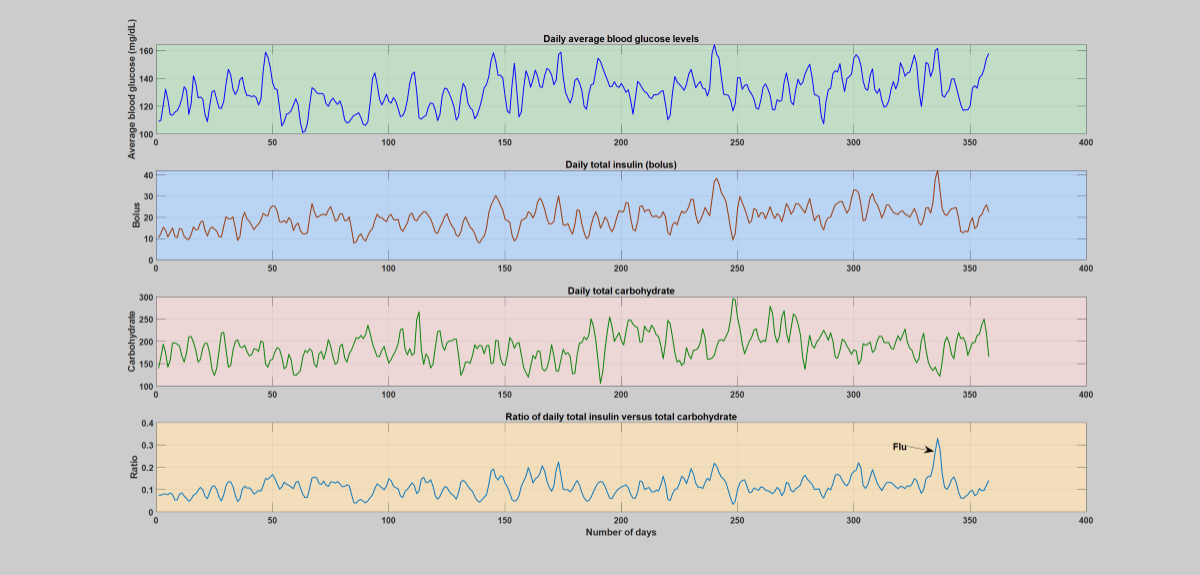
Daily analysis of the third infection case (flu). The figure depicts variation of average blood glucose levels, total insulin (bolus), total carbohydrate, and total insulin-to-total carbohydrate ratio. The operating point of the patient’s insulin-to-carbohydrate ratio had dramatically shifted and raised above the regular or normal days and topped around 0.4 upon midinfection week.

**Figure 10:**
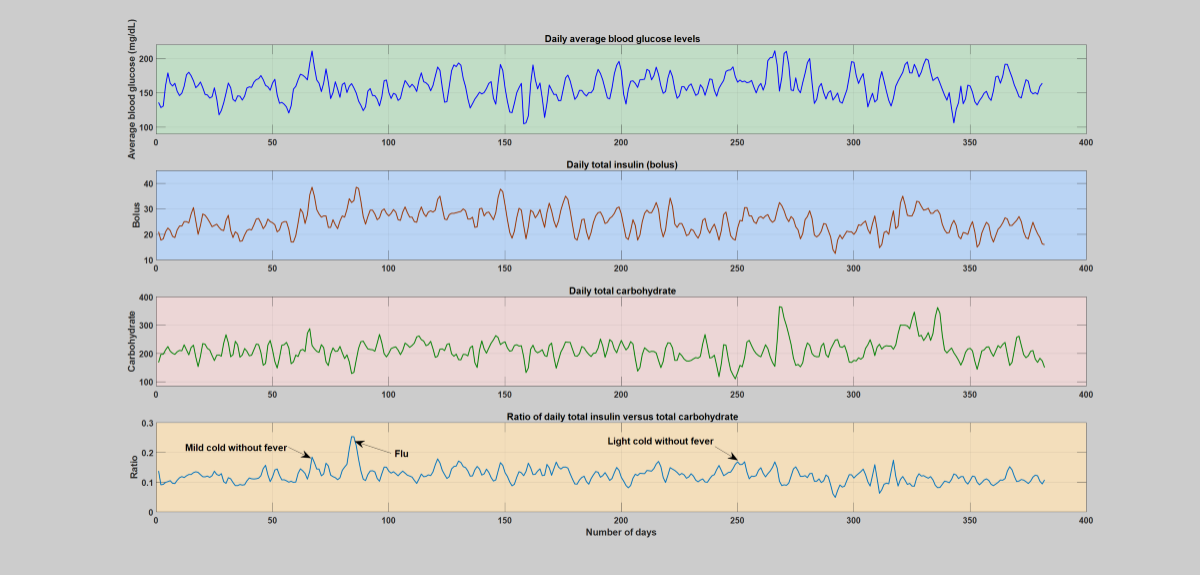
Daily analysis of the fourth infection case (mild common cold without fever, light common cold without fever, and flu). The figure depicts variation of average blood glucose levels, total insulin (bolus), total carbohydrate, and total insulin-to-total carbohydrate ratio. The operating point of the patient’s insulin-to-carbohydrate ratio had dramatically shifted and raised above the regular or normal days and reach a top around 0.28 upon midinfection week. A light common cold without fever seems to not significantly affect the operating point.

**Figure 11:**
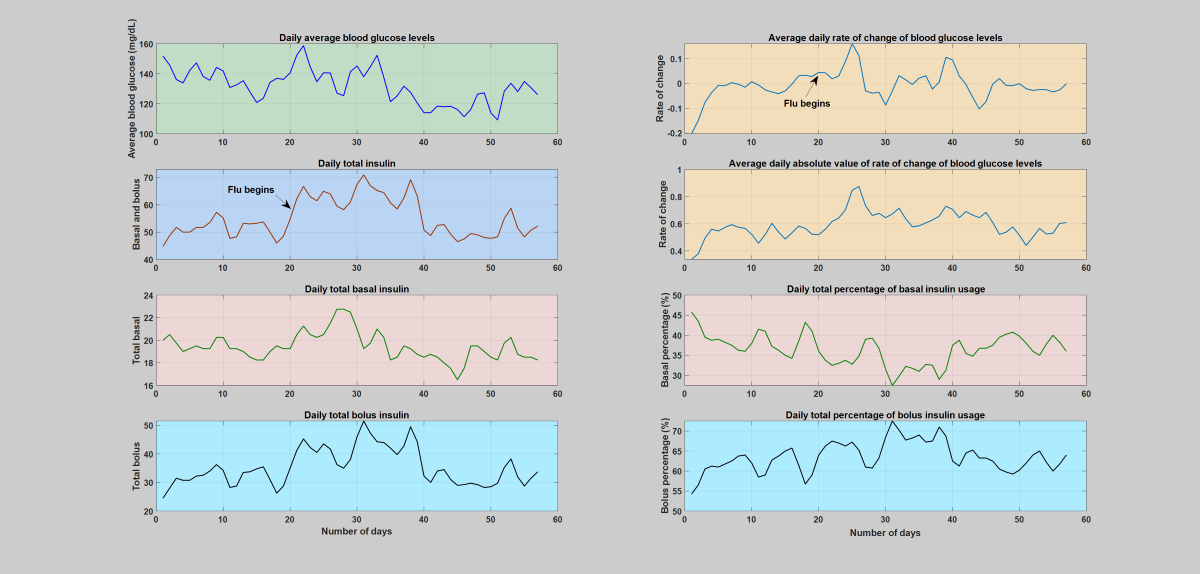
Daily analysis of the fifth infection case (flu). The figure depicts variation of average blood glucose levels, total insulin including both bolus and basal insulin, daily average rate of change of CGM and, absolute value of rate of change of CGM (computed based on CGM direction from the pump information), percentage of basal and bolus per total insulin units. As can be seen, as a result of the ongoing infection incidence, there is clear and dramatic rise in the amount of insulin, while blood glucose levels remain elevated.

Hourly and daily analyses were conducted by analyzing the deviations incurred on the key diabetes parameters, blood glucose levels, insulin, carbohydrate, and the insulin-to-carbohydrate ratio as a result of infection incidence in contrast to the whole patient year. The comparison was carried out based on the smoothed version of the data, that is, 2 days window moving-average filter. Similar to the weekly analysis, the infection-induced shift of the blood glucose dynamics, that is, higher glucose production and increased insulin resistance, is clearly shown in both the daily and hourly analyses. As can be seen in Figures 6-11, the insulin-to-carbohydrate ratio of the patient has drastically shifted to a higher value to account for the effect of increased glucose production and insulin resistance (see Multimedia Appendix 3 for a detailed plot of the hourly analysis in all the infection cases). In all of these cases, the insulin-to-carbohydrate ratio increases from the usual values of 0.05 to 0.2 during the normal period to higher values reaching 0.6, depending on the degree of severity of the infection incidence, type of pathogens involved, and the individual immunity.

#### Kernel Density Estimation–Probability Distribution

**Figure 12:**
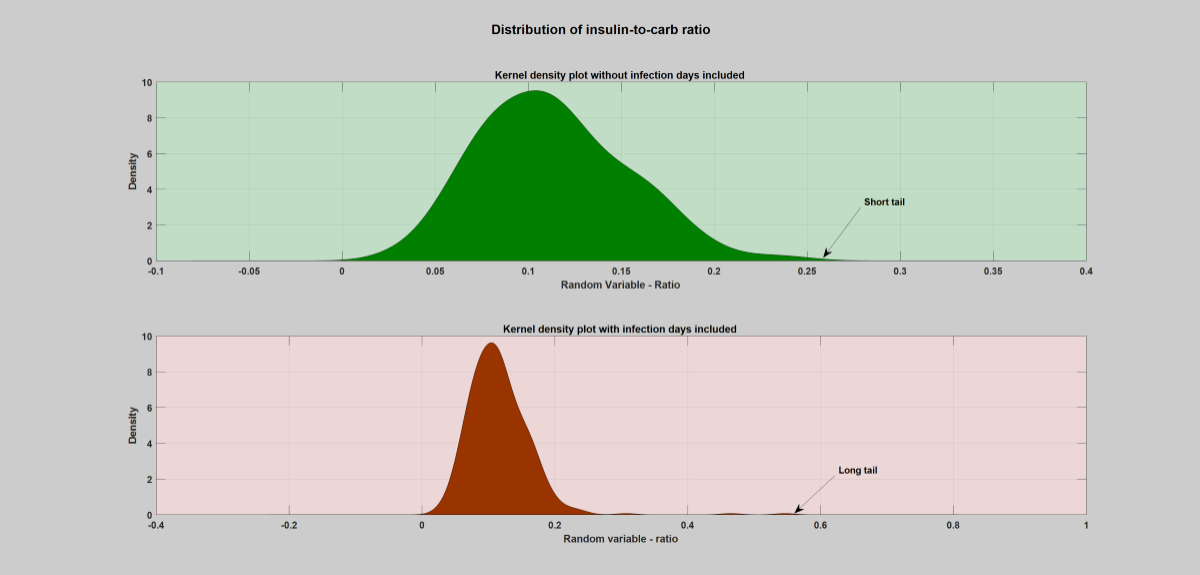
Univariate kernel density estimation of a patient year using the daily insulin-to-carbohydrate ratio. As can be seen from the tail of the distribution, during regular or normal days (the green shaded region), the yearly distribution of the patient’s insulin-to-carbohydrate ratio lies within the values of 0.005 and 0.2. However, during infection incidence (the red shaded region), there is a clear deviation in the tail of the distribution, where the values reaches around 0.58.

**Figure 13:**
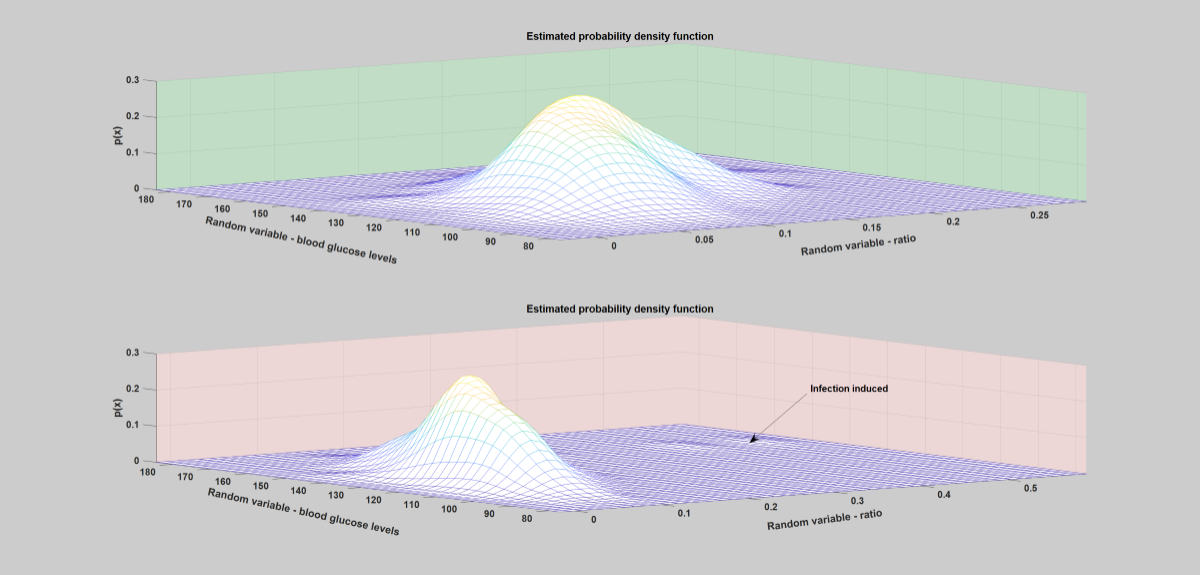
Bivariate kernel density estimation of a patient year using both the daily average blood glucose levels and insulin-to-carbohydrate ratio. As can be seen from the bivariate distribution, during regular or normal days (the top light green figure), the distributions are concentrated around the high density regions. However, during infection incidence (the lower figure), there is a clear bump far from the high density regions.

Kernel density was estimated to study and characterize the nature, shape, and degree of severity of the deviations incurred due to infection incidence by analyzing the probability distribution of the individual key parameters of the blood glucose dynamics. A univariate and bivariate kernel density estimation based on the insulin-to-carbohydrate ratio and blood glucose levels was carried out on the yearlong data, as shown in Figures 12 and 13 (a detailed plot for all the infection cases, both hourly and daily, can be found in Multimedia Appendix 1). As can be seen from the figures, the infection incidence has brought a significant change in the probability distribution. However, the nature, shape, and degree of outlierness depend on the type of pathogen involved, severity of infection, and individual immunity.

## Discussion

### Principal Findings

Presently, in relation to people’s mobility and travel, there is a growing concern regarding an infectious disease outbreak. Such an incident can be a menace to our global health security, which calls for early detection and immediate response. Thus, there is a growing need for new approaches and technologies to upgrade the existing surveillance system for early detection of emerging infectious diseases [1]. Existing disease surveillance systems detect the incidence of outbreaks long after the incidence of the first symptoms. Therefore, the purpose of this study was to demonstrate how people with type 1 diabetes can assist in outbreak detection and further to shed light upon the possibility of assisting the individual during such an incident.

The advancement and omnipresence of smartphones, IoT devices, wearables, and sensors have enabled individuals to easily self-record health-related events often for self-tracking or self-managing their disease [5,6,53]. People with diabetes self-record detailed information including blood glucose levels, diet and insulin intake, physical activity, medication, and other parameters. The presence of such large self-recorded health data presents an opportunity to be used as a secondary source of information for other purposes such as digital epidemiology and decision support applications. According to recent reports, the use of personal health information or self-collected data could mitigate the possibility of detecting infection incidence during the presymptomatic stage (improved sensitivity and timeliness), specifically during the incubation period, of which most of the current systems neglect from their process [13]. Our findings demonstrated that upon infection incidence, there is a dramatic shift in the operating point of the individual’s blood glucose dynamics, which clearly violates the usual norm of blood glucose dynamics. During regular or normal days, blood glucose levels usually decrease when there is a significant increase in insulin injection and reduction in carbohydrate consumption. However, in all of the infection cases we analyzed, compared with the preinfection and postinfection weeks, the following were noticed:

- Blood glucose levels were elevated by an average of 6.1% and 16% over the preinfection and postinfection weeks, respectively.
- Insulin injection (bolus) increased by 42% and 39.3% over preinfection and postinfection weeks, respectively.
- Carbohydrate consumption was reduced by 19% and 28.1% compared with preinfection and postinfection weeks, respectively.
- The insulin-to-carbohydrate ratio increased by 108.7% on average in all cases.

In general, all of these findings confirm that during infection incidence, blood glucose levels are elevated despite injecting higher amounts of insulin and reduced carbohydrate consumption. The identified changes are quite significant anomalies compared with the regular or normal days and could potentially be detected with a dedicated personalized (individualized) computational health model. Various algorithms that span from prediction models to anomaly detection algorithms can be investigated to detect such infection-induced changes in blood glucose dynamics. Apart from the potential use of these findings in personalized digital infectious disease detection systems, it could also be used for decision support in self-management during infection and illness. As presented earlier, during the course of infection, individuals with diabetes usually struggle with severe hyperglycemia. Managing blood glucose levels during infection incidence is not an easy task, given the fact that it is caused by a mixed effect of both patient-controllable and patient-uncontrollable parameters. The patient can only estimate the disturbance caused by the amount of carbohydrate consumption, insulin injection, and physical activity load, which is not the case during infection incidence. Apart from these known major factors, that is, patient-controllable parameters, there is an underlying and unknown disturbance caused by the patient’s uncontrollable parameters, such as counterregulatory hormones (CRHs), as a result of infection incidence. This unknown disturbance mainly increases glucose production from the liver and reduces insulin sensitivity. To this end, people with type 1 diabetes face a very difficult challenge to estimate the necessary amount of insulin for a given amount of carbohydrate consumption. In this regard, providing real-time decision support could reduce the burden during such a crisis. One possible approach could be characterizing the effect of different pathogens on blood glucose dynamics, mainly on insulin resistance and its sensitivity change over the course of infection. However, a large set of infection-related self-recorded data need to be analyzed for investigating how each pathogen affects the key parameters of blood glucose dynamics during the entire course of infection. This requires collecting and analyzing infection-related data, and estimating the overall changes each pathogen could bring on insulin sensitivity during the course of infection. To this end, the presented result reflects a promising result that can be geared toward decision support during infection or illness. For example, the change in insulin–to-carbohydrate ratio can be used to provide general information related to each pathogen on what to expect, such as the percentage of insulin resistance during the first days, in the middle, and at the final days of the infection.

### Infection-Induced Shift of Operating Point in Blood Glucose Dynamics

During infection incidence, people with diabetes usually struggle with severe hyperglycemia and critical hypoglycemia if not properly managed. However, during regular or normal days, the patient can manage the incidence of hyperglycemia, which is mostly diet-induced, by properly controlling the patient-controllable parameters, for example, amount of carbohydrate consumption, insulin injection, and performing balanced physical activity or exercise. Yet, during infection incidence, it turns out to be very difficult to manage the hyperglycemia incidence due to the fact that it is caused by a mixed effect of both patient-controllable and patient-uncontrollable parameters. The patient’s uncontrollable parameters define the action of hormonal effects such as CRH induced by either physiological stress or emotional stress. The hormonal effect is two-sided, which is a higher glucose production from the liver and inhibiting insulin production and reducing sensitivity [54,55]. A detailed study conducted by Waldhausl et al [56] demonstrated the significant effect of stress hormones on the production of glucose and insulin resistance. The study was conducted by infusing different stress hormones to investigate the effect of exposure to these hormones on blood glucose response [55]. The extent and degree of hyperglycemia events and insulin resistance during infection incidence directly correlate with the type of pathogen, the type of hormone involved and the severity of the infection [37,38,55]. Generally, the phenomenal effect of infection incidence on blood glucose dynamics in people with diabetes can be simply described using the following relationships:



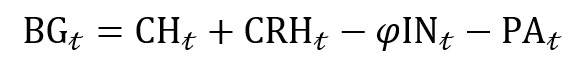



Where *φ* is an insulin sensitivity factor, BG is the blood glucose level, CH is the amount of carbohydrate consumption, IN is the amount of insulin injection, PA is the amount of physical activity session or exercise load, and CRH is the effect of CRHs. The equation depicts the phenomena that occur during infection incidence, where blood glucose levels are raised by the action of both patient-controllable parameters (CH) and patient-uncontrollable parameters (CRHs, such as cortisol and adrenalin). Thus, consumption of any regular diet in an individual can induce severe hyperglycemia due to the added effect of glucose production from the liver as a result of the CRH effect [55]. For this reason, the patient is expected to reduce the amount of carbohydrate intake to a certain extent to optimally manage the hyperglycemia crises and at the same time avoiding any critical hypoglycemia incidences (for more information, see Multimedia Appendix 1). By the same token, blood glucose levels can be lowered to euglycemia by the patient-controllable parameters (insulin [IN] and physical activity session or exercise load [PA]). However, due to the change in insulin sensitivity, the action of insulin is reduced (*φ* is affected by infection incidence), and the patient is expected to deliver more insulin injections to counterbalance the effect of insulin resistance [57]. According to our results, all these scenarios are reflected in the individual’s blood glucose dynamic infected with flu (influenza), where a dynamic shift occurred from the usual operating point of the blood glucose dynamics. There are elevated blood glucose levels, despite injecting a higher amount of insulin and consuming less carbohydrate than the regular or normal days. These characteristics are clearly demonstrated on the shift incurred on the individual’s insulin–to-carbohydrate ratio as compared with the regular or normal days. Therefore, blood glucose, amount of injected insulin, diet intake, and insulin-to-carbohydrate ratio and other supporting physiological parameters such as body temperature and blood pressure can be exploited to develop a personalized health model for detecting infection incidence among people with type 1 diabetes. Given the similarity, this result can also be translated to other types of diabetes, such as people with type 2 diabetes. It is worth mentioning that apart from infection incidence, other factors such as emotional stress could also result in similar variable episodes of elevated blood glucose levels [17]. This can obviously impact the detection performance of the model. However, our results based on yearlong patients’ data demonstrated that the use of carbohydrate consumption, insulin injections, and insulin-to-carbohydrate ratio along with the blood glucose could solve this confounding nature. Moreover, acute emotional stress, other than the chronic ones, might have less influence on one’s meal appetite compared with infection incidence to skew the insulin-to-carbohydrate ratio [17].

### Relevance of the Data

The informational values of the data, availability of the data, and cost of the data are the 3 key metrics necessary to evaluate the relevance of new surveillance data for a digital infectious disease detection system [58]. The informational value of the data assesses how informative the data are to facilitate the detection or characterization of infectious disease outbreaks. In this regard, the surveillance data must clearly indicate the absence or presence of infections either on an individual or population level or both in a timely manner. Furthermore, the rate of false alarms derived from the data is an important factor that dictates the acceptability of the surveillance data, which is in turn governed by the signal-to-noise ratio defining the signal’s strength depicting the infection period as compared to the regular or normal period (baseline data) [58]. In this regard, our results demonstrated that the infection-induced signal exhibits high discriminative power from the baseline (normal or regular) patterns. The availability of surveillance data is another crucial indicator for screening potential types of data, which needs to be addressed [58]. In this regard, given the widespread and ubiquitous nature of mobile apps, and different sensors, people with type 1 diabetes collect far more data than ever. For example, many people with type 1 diabetes use continuous glucose monitors and insulin pumps, which are predicted to grow further in terms of both quality and quantity of data in the coming years. The most crucial challenge in this direction includes issues related to security, privacy, and confidentiality of user data if there is a necessity to collect user data into a central server than deploying the detection algorithm on the user’s own mobile device. The cost of data delineates the associated cost in relation to acquiring the data in question, including the cost incurred for realizing the data collection system [58]. In this regard, the individual’s self-recorded data are solely collected for their own use and used as a secondary source of information for disease surveillance purposes. Providing tailored and valuable feedback to the individual patient might further motivate them to participate on a large scale (for further details, see the section Ethical and Motivational Challenges).

### Framework of a Personalized Digital Infectious Disease Detection System

**Figure 14:**
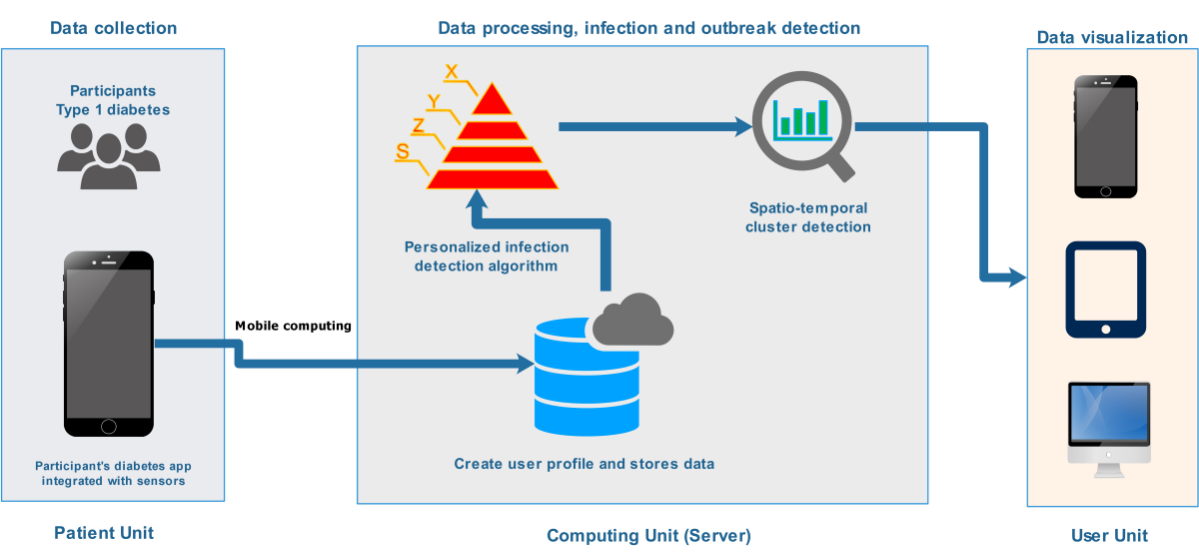
The Proposed System Architecture.

Epidemic intelligence encompasses activities directed toward early detections, verification, and assessment of potential public health threats to notify and recommend necessary measures for the concerned bodies regarding the ongoing situation [56]. Early detection systems such as Google Flu Trends and other existing systems have certain limitations because they do not have the mechanisms to identify or track individual cases through diagnosis or screening based on a personalized health model. This limitation has a major impact and certainly introduces bias in disease outbreak prediction. Currently, a personalized health model, which resembles the way clinicians and epidemiologists classify an individual as normal, suspected, or confirmed case, for screening and case detection doesn’t exist [58]. Having a personalized health model can provide information for both individual health-related decision support purposes and at the same time can be used for tracking infectious disease outbreaks among the public. The results of this study demonstrated that commencement of infection in people with type 1 diabetes significantly alters the individual blood glucose dynamics, and such a change can potentially be detected through modeling of the individual blood glucose dynamics. Moreover, incorporating various physiological parameters, for example, heart rate and body temperature, to a personalized health model will further enable the capture of infection incidence as early as possible, that is, incubation period. Therefore, the development of a personalized health model–based digital infectious disease detection system is vital for the success of next-generation public health surveillance systems. The data sources and signal exploited, outbreak detection algorithms employed, clustering approaches, and visualization techniques used to play a central role in any digital infectious disease detection systems by determining its accuracy (sensitivity) and timeliness (lead time) [56]. On the basis of the kind of data sources and signals exploited, infectious disease surveillance systems can be generally grouped into an indicator-based and event-based system [56,59,60]. Event-based systems mainly rely on unstructured data collected through formal or informal sources and is characterized by quick detection, reporting, and assessments of public health events, including clusters of disease [56,60]. On the other hand, indicator-based systems mainly use structured data, which are collected following a standard case definition and is characterized by routine reporting of disease cases [56,60]*.* The proposed system [26,61], as shown in Figure 14, is categorized under event-based digital infectious disease detection systems, where the events are grouped under microevents and macroevents [56]*.* Under the umbrella of these events and the proposed system in general, a framework of several components such as infection detection algorithms (how to develop an algorithm to detect infection incidence at the individual level); clustering algorithms (how to group the infected individuals to form a cluster); visualization techniques (how to report and display the detected outbreak incidence) and further ethical and motivation challenges are briefly discussed below.

#### Microevent: Individual-Level Detection of Infection Incidence

**Figure 15:**
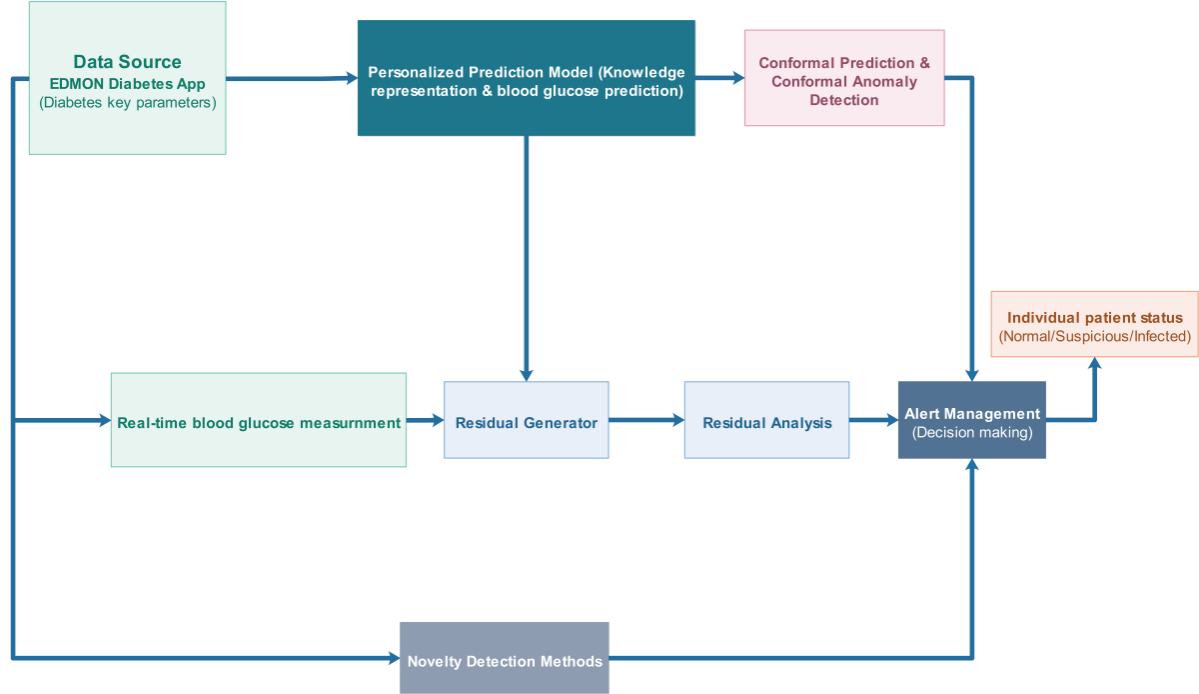
The proposed personalized infection detection algorithms for detecting microevents (incidences of infections) in people with type 1 diabetes. These approaches are alternative means of achieving the same objective, which is detecting infection incidences.

The detection of microevents as the name suggests is carried out at an individual level by tracking the individual’s diabetes profile including blood glucose levels, amount of insulin injections, carbohydrate consumption, physical activity or exercise sessions, and others. The presence of elevated blood glucose levels despite injecting higher amounts of insulin and consumption of less carbohydrates is regarded as a marker of an event of infection incidence and hence can be defined as a microevent for the event-based digital infectious disease detection system. Detecting the incidence of these kinds of deviation from the usual norm of blood glucose dynamics requires a proper personalized health model, which can learn from past history of the patient and judge whether the information conforms with the usual trend. Hence, the proposed personalized health model for detecting these types of microevents incorporates 3 components: a data source, personalized infection detection algorithm, and alarm management module, as shown in Figure 15. As can be seen from the figure, the personalized infection detection algorithm can be modeled using either a prediction model–based approach or a novelty or anomaly detection–based approach.

##### Data Sources and Input

The patient unit is a mobile health app, as shown in Figure 14, which integrates data from different sensors and wearables that record key diabetes parameters, such as blood glucose levels, insulin dosage, diet, physical activity, and other optional physiological parameters including body temperature, heart rate, blood pressure, and others [26,61]. The app is also expected to record the geographical location of the individual along with the time of data registration. For example, one way of estimating user location can be carried out based on global positioning system (GPS) information from the mobile phone during data registration [61]. The geographical location of the user can be the geographical coordinates of longitude and latitude [62], postal code address [63], or any local reference coordinates.

##### Personalized Infection Detection Algorithm

Detection of microevents can be carried out using individual self-recorded historical data based on a personalized health model, that is, either a prediction model [64,65] or novelty detection algorithms [66-68], as shown in Figure 15. The prediction model–based algorithm requires learning the individual blood glucose dynamics for accurate prediction, and for the purpose of detecting the microevents, it can be implemented as either a residual-based [69-72] or a conformal prediction–based approach [73-80]. In a similar fashion, novelty detection–based algorithms can be other alternatives for detecting novel microevents relying on either supervised, semisupervised, and unsupervised approaches [4,66,67,81]. Different categories of novelty detection approaches could be exploited for detecting infection-induced deviations in blood glucose dynamics, including approaches based on statistical techniques [68], prediction, density [82-85], distance [67,86], classification or domain [4,62,63,87-92], clustering [62,93], and ensemble [67,68,80-82,85,86,92-95]*.*

##### Alarm Management (Decision Making)

The alarm management module accepts the score computed by the personalized infection detection algorithm as input and evaluates the degree of severity of the infection incidence. The severity is evaluated based on the degree of abnormalities of the anomalies score, and a label could be assigned to the individual patient status as normal (0), suspicious (−1), and infected (1). For example, a rule-based fuzzy logic with membership functions of infected, normal, and suspicious can be used to assign the label indicating the severity of the infection incidence using the anomaly score. The output from the alarm management will be directly fed to the cluster detection analysis, which is used to detect a group of patients based on geography (space) and time so as to revel if there is any ongoing infectious disease outbreak.

#### Macroevents: Population-Level Infectious Disease Outbreak Detection

##### Cluster Detection Mechanism

Cluster detection is defined as the process of identifying a group of infected individuals with similar spatial, temporal, or spatio-temporal attributes [96]. A spatial cluster analysis only considers a patient’s geographical location, and a temporal cluster analysis considers only the time aspect of the events. However, a spatio-temporal cluster analysis is conducted to look for aberrant patterns and detect a cluster of infected people within a specified geographical region and predefined timeframe [96,97]. The analysis of space time clusters is carried out based on a couple of steps: geocoding and identification, which transforms the patient address into meaningful coordinates and detecting the clusters based on the transformed location and time. A space time cluster analysis is the most favored approach when it comes to early detection of an infectious disease outbreak. A space time cluster analysis can be designed by performing a spatial analysis first and then superimposing the temporal aspect [97]. Regarding the proposed system, the input to the space time cluster detection analysis consists of the individual patient status from alarm management, user location, and time of data registration [26]. The status of the individual patient at any time can be normal (0), infected (1), or suspicious (−1), which comes from alarm management. The user’s geographical location can be geographical coordinates of longitude and latitude [98], postal code address [99], or any local reference coordinates. Estimation of user location can be carried out using GPS information from the user’s mobile phone, which can be accessed during each data registration. The time aspects depend on the requirement of detection frequency and can be set to either an hourly or daily window. One optimal approach could be tracking the individual during each hour of the day for any statistically significant deviations and performing a concluding analysis at the end of each day based on the daily analysis. Various algorithms have been implemented in the literature, including the density-based clustering algorithm, Bayesian spatial scan statistics, K-NN with Haversian distance (K-nearness), cumulative summation, space time scan statistics, space time permutation scan statistic, and space scan statistics [96,97,100], which can be further tested and adopted. The most important challenge is the sparsity of the data set considering the small proportion of people with type 1 diabetes that can be under surveillance over a large region. Therefore, it is necessary to adopt these cluster detection techniques to overcome data sparsity and produce acceptable detection accuracy. In the proposed system, the detected clusters, if there is any, can be displayed and viewed based on real-time and interactive data visualization tools.

##### Data Visualization

Data visualization is a mechanism by which detected clusters of disease outbreaks, if there is any, are presented to the responsible bodies for quicker public health actions and responses. Generally, such a visualization tool could report outbreaks of epidemic cases for investigation and follow-up, and it could also report the duration of the epidemic (timing), degree of severity of the epidemic, and the region under threat. In the literature, there are various implemented visualization tools and visual displays with regard to disease outbreak detection systems, including ArcGIS, Google map API, TwiInfo, OpenStreetMap, and JFreeChart, and display mechanisms such as maps, time series, graphs, and color indicators [96]. These visualization tools and display mechanisms can be further tested and adopted in the proposed system. The real-time health status of an individual from the ongoing tracking could be accessible to the end user and can be displayed in a stand-alone software app based on smartphones, tablets, and computers or a dedicated website [26]. Generally, both the data providers (participants) and the general population could benefit from the system in the sense that they can take actions needed to avoid being infected. Moreover, the individual patient could also receive analysis and feedback from the system to learn the situation, such as the degree and severity of deviation of different parameters, including blood glucose, insulin, diet, and insulin-to-carbohydrate ratio, along with their trend as compared with the noninfection period.

#### Ethical and Motivational Challenges

The implementation of a digital infectious disease detection system based on self-recorded data poses serious challenges that require special attention, such as user privacy and security, data confidentiality, user acceptance, and motivations [26,101], especially during data collection, transmission, and data storage [102,103]. Personal health–related data are sensitive, and the data collection, transmission, and data storage procedure need to follow the standards and regulations provided by the major governing bodies, such as General Data Protection Regulation (GDPR) and Health Insurance Portability and Accountability Act (HIPAA) [104,105]. This includes privacy-preserving mechanisms such as pseudonymization and anonymization to meet the necessary data compliance requirement along with user informed consent [102,103]. According to GDPR, the deidentification procedure is one of the recommended anonymization standards to preserve data confidentiality [104,105]. Moreover, from the technology perspective, it is necessary to look for a robust mechanism to ensure that user privacy and security are respected during data collection, transmission, and storage, as this is highly critical for successful acceptance of the proposed system [26,106]. One such alternative is to look for the possibility of deploying the infection detection algorithm (app logic) on the user (client) mobile device terminal to avoid transmission of patient data to a central server, where only the timely computed infection status of the patient will be sent to the central server for further cluster detection processing. However, this choice requires further feasibility studies to determine the cost, especially in terms of power constraints related to the mobile device terminal, since the detection algorithms need to continuously run in the background to compute the individual’s infection status, at the most each hour of the day [26]. In addition, users might also lack willingness to adopt a new technology or system for various reasons ranging from lack of trust, lack of motivation, lack of perceived usefulness, and ease of use [26,101]. However, these challenges can be mitigated by properly buying user trust by developing state-of-the-art technology for preserving privacy, security, and confidentiality of the user and addressing factors that enhance user motivation, including usability knowledge, simplicity and ease of use, reduced time and frequency of interaction with the system, incentives, and others [101].

### Conclusions

The relationship between infection incidents and elevated blood glucose levels has been known for a long time. People with type 1 diabetes often experience prolonged episodes of elevated blood glucose levels as a result of infection incidence. Despite the fact that patients increasingly gather data about themselves, there are no solid findings on how to use such self-recorded data as a secondary source of information for other purposes, such as self-management–related decision support during infection incidence and digital infectious disease detection systems. We presented the effect of infection incidence on key parameters of the blood glucose dynamics along with the necessary framework to exploit the information for realizing a digital infectious disease detection system and further shed light on the possibility of assisting individuals during infection-related blood glucose management crises. The results demonstrated that despite tight blood glucose control, blood glucose level is still elevated during infection incidence. The analysis shows that infection incidences have a significant impact on blood glucose dynamics as compared with the other patient-uncontrollable factors. All of these findings indicate that blood glucose levels were elevated despite a higher amount of insulin injection and reduced carbohydrate consumption, which are quite significant changes that could possibly be detected through personalized modeling that spans from prediction models to anomaly detection algorithms. However, further large-scale studies are required to strengthen the findings. Moreover, future research should investigate the possibility of improving detection time and disease characterization. Early detection, that is, during the incubation period, is a critical component of any outbreak detection system and therefore needs to be improved by analyzing how various features of CGM can be used in context with other parameters, such as diet, insulin, and physical activity data. For instance, different individuals with type 1 diabetes often reported the experiences of an elevated episode of blood glucose levels before the onset of the first symptoms. Disease characterization involves determining the type and nature of pathogens that cause the infection, which is an important component of outbreak reporting. The extent and degree of the impact of infection incidence on blood glucose dynamics are highly correlated with the disease pathogens involved. In this regard, carefully analyzing a large-scale self-recorded data set containing several infection incidences (different pathogens) could characterize them based on their effect on blood glucose dynamics. Generally, we foresee that these findings can benefit the efforts toward building next-generation digital infectious disease surveillance systems and provoke further thoughts in this challenging field.

## Appendix

Multimedia Appendix 1 Comparative analysis of parameters of blood glucose dynamics with and without infection incidences.

Multimedia Appendix 2 Analytical plot of the normal/regular patient years.

Multimedia Appendix 3 Analytical plot of the patient years with acute infection.

## Abbreviations

CGM: continuous glucose monitoring
CRH: counterregulatory hormone
GDPR: General Data Protection Regulation
GPS: global positioning system
IoT: Internet of Things
SMBG: self-monitoring of blood glucose
