# Supplementary material for: Toward Detecting Infection Incidence in People With Type 1 Diabetes Using Self-Recorded Data (Part 1): A Novel Framework for a Personalized Digital Infectious Disease Detection System
Source: J Med Internet Res. 2020 Aug 12;22(8):e18911. doi: 10.2196/18911 (PMC7450374; doi:10.2196/18911)
Supplement: Multimedia Appendix 1 [file jmir_v22i8e18911_app1.docx]

# **Appendix 1: Comparative Analysis of Parameters of Blood Glucose Dynamics with and without Infection Incidences**

## **Pre-Infection, Infection, and Post-Infection Week Analysis**

The analysis is done based on the daily average blood glucose levels, total insulin including both bolus and basal whenever possible and total carbohydrate consumptions. As shown in the **Table 1** and **Figure 1-5**, the analysis has demonstrated that during all the infection incidences,

- Blood glucose is too resistant to drop and remains elevated in all the cases.
- There is high injection of insulin compared to the pre and post infection week.
- Carbohydrate is significantly reduced as compared to the pre and post infection week.

Detailed demonstration is presented in the following section.

**Table 1**: Mean and standard deviation of BG levels, total insulin (bolus), and total carbohydrate during the pre-infection week, infection week and post-infection week.

| **The first case of infection (flu)** | | | |
| --- | --- | --- | --- |
| **Parameter** | Pre-infection week  ($Mean (SD)$) | Infection week  ($Mean (SD)$) | Post-infection week ($Mean (SD)$) |
|  | **Daily** | | |
| Blood Glucose (mg/dl) | $130.74 (16.89)$ | $141.95 (14.37)$ | $119.16 (7.39)$ |
| Total insulin (bolus) | $23.39 (4.91)$ | $35.30 (6.11)$ | $21.32 (4.61)$ |
| Carbohydrate (grams) | $241.11 (57.27)$ | $178.80 (65.69)$ | $241.18 (37.63)$ |
|  | **Hourly** | | |
| Blood Glucose (mg/dl) | $134.24 (32.67)$ | $147.38 (36.89)$ | $129.77 (39.13)$ |
| Total insulin (bolus) | $0.99 (1.87)$ | $1.51 (2.32)$ | $0.89 (1.77)$ |
| Carbohydrate (grams) | $10.16 (16.50)$ | $7.32 (13.76)$ | $10.20 (15.84)$ |
| **The second case of infection (flu)** | | | |
|  | **Daily** | | |
| Blood Glucose (mg/dl) | $143.01 (19.53)$ | $155.36 (21.99)$ | $126.17 (11.70)$ |
| Total insulin (bolus) | $28.07 ( 8.85)$ | $41.07 (9.44)$ | $25.36 (6.93)$ |
| Carbohydrate (grams) | $190.14 (43.93)$ | $161.14 (58.43)$ | $214.57 (34.66)$ |
|  | **Hourly** | | |
| Blood Glucose (mg/dl) | $147.51 (40.75)$ | $162.32 (39.49)$ | $130.85 (36.49)$ |
| Total insulin (bolus) | $1.14 (1.92)$ | $1.68 (2.48)$ | $0.98 (1.94)$ |
| Carbohydrate (grams) | $8.3517 (14.68)$ | $6.88 (14.25)$ | $8.39 (14.56)$ |
| **The third case of infection (flu)** | | | |
|  | **Daily** | | |
| Blood Glucose (mg/dl) | $136.93 (18.58)$ | $144.12 (20.30)$ | $134.18 (11.96)$ |
| Total insulin (bolus) | $20.08 ( 5.44)$ | $31.50 (10.84)$ | $22.83 (3.86)$ |
| Carbohydrate (grams) | $178.0 (45.87)$ | $144.83 (37.63)$ | $195.83 (42.59)$ |
|  | **Hourly** | | |
| Blood Glucose (mg/dl) | $143.30 (40.51)$ | $149.84 (32.90)$ | $139.77 (39.61)$ |
| Total insulin (bolus) | $1.00 (1.77)$ | $1.50 (2.17)$ | $0.93 (1.62)$ |
| Carbohydrate (grams) | $7.71 (14.31)$ | $6.48 (12.82)$ | $7.28 (13.42)$ |
| **The fourth case of infection (flu)** | | | |
|  | **Daily** | | |
| Blood Glucose (mg/dl) | $157.74 (31.12)$ | $161.34 (19.88)$ | $138.57 (19.83)$ |
| Total insulin (bolus) | $24.43 (5.26)$ | $32.14 (7.01)$ | $29.29 (5.22)$ |
| Carbohydrate (grams) | $199.06 (53.45)$ | $167.04 (44.94)$ | $226.07 (18.23)$ |
|  | **Hourly** | | |
| Blood Glucose (mg/dl) | $151.57 (51.15)$ | $157.68 (52.43)$ | $142.16 (47.79)$ |
| Total insulin (bolus) | $0.96 (2.60)$ | $1.39 (2.97)$ | $1.19 (3.24)$ |
| Carbohydrate (grams) | $8.38 (20.25)$ | $6.78 (18.52)$ | $9.78 (22.57)$ |
| **The fifth case of infection (flu)** | | | |
|  | **Daily** | | |
| Blood Glucose (mg/dl) | $135.21 (14.58)$ | $139.88 (15.54)$ | $122.87 (14.49)$ |
| Insulin (bolus) | $32.80 (4.59)$ | $40.37 (8.31)$ | $33.36 (7.94)$ |
| Insulin (basal) | $19.20 (1.21)$ | $20.42 (2.06)$ | $18.68 (1.56)$ |
| Total Insulin | $52.33 (5.14)$ | $61.21 (8.26)$ | $52.46 (8.47)$ |
|  | **Hourly** | | |
| Blood Glucose (mg/dl) | $134.23 (34.16)$ | $144.09 (44.25)$ | $122.12 (35.99)$ |
| Insulin (bolus) | $1.36 (2.58)$ | $1.76 (2.82)$ | $1.45 (2.63)$ |

## The First Case of infection (flu)

1. Comparison of Blood Glucose levels during pre-infection week, infection week and post-infection week. As can be seen, the blood glucose is elevated during the infection week as compared to the pre and post-infection week.

1. Comparison of Insulin (bolus) intake during pre-infection week, infection week and post-infection week. As can be seen, the amount of insulin (bolus) intake is elevated during the infection week as compared to the pre and post-infection week.

1. Comparison of Carbohydrate (grams) intake during pre-infection week, infection week and post-infection week. As can be seen, the amount of Carbohydrate (grams) intake is significantly reduced during the infection week as compared to the pre and post-infection week.
2. **Table 2**: Mean Percentage Change between pre-infection week versus infection week and post-infection week and infection week. For further reference, see **Table 1** Above.

| **Parameters** | **Pre-infection Vs. Infection week** | **Post-infection week Vs. Infection week** |
| --- | --- | --- |
| **Blood Glucose** | **8.57%** | **19.12%** |
| **Insulin (bolus)** | **50.93%** | **65.59%** |
| **Carbohydrate** | **-25.84%** | **-25.87%** |
| **Hourly** |  |  |
| **Blood Glucose** | **9.79%** | **13.57%** |
| **Insulin (bolus)** | **52.53%** | **70.00%** |
| **Carbohydrate** | **-27.95%** | **-28.24%** |

**Figure 1**: Analysis of pre-infection week, infection week, and post-infection week based on the first patient year. Figure (a) depicts the blood glucose levels during these weeks. Figure (b) depicts the amount of insulin (bolus) injected during these weeks. Figure (c) depicts the amount of carbohydrate consumed in grams during these weeks. Table 2 shows the mean percentage change between these weeks. In all the figures, the asterisk shows the mean value and the red line depicts the median value for the week.

## The Second Case of Infection (flu)

1. Comparison of Blood Glucose levels during pre-infection week, infection week and post-infection week. As can be seen, the blood glucose is elevated during the infection week as compared to the pre and post-infection week.

1. Comparison of Insulin (bolus) intake during pre-infection week, infection week and post-infection week. As can be seen, the amount of insulin (bolus)intake is elevated during the infection week as compared to the pre and post-infection week.

1. Comparison of Carbohydrate (grams) intake during pre-infection week, infection week and post-infection week. As can be seen, the amount of Carbohydrate (grams) intake is significantly reduced during the infection week as compared to the pre and post-infection week.
2. **Table 3**: Mean Percentage Change between pre-infection week versus infection week and post-infection week and infection week. For further reference, see **Table 1** Above.

| **Parameters** | **Pre-infection Vs. Infection week** | **Post-infection week Vs. Infection week** |
| --- | --- | --- |
| **Blood Glucose** (mg/dl) | 8.63**%** | 23.13**%** |
| **Insulin (bolus)** | 46.31**%** | 61.94**%** |
| **Carbohydrate** (grams) | -15.25**%** | -24.90**%** |
| **Hourly** |  |  |
| **Blood Glucose** (mg/dl) | **10.04%** | 24.05**%** |
| **Insulin (bolus)** | **47.13%** | 70.87**%** |
| **Carbohydrate** (grams) | -17.59**%** | -18.0**%** |

**Figure 2**: Analysis of pre-infection week, infection week, and post-infection week based on the second patient year. Figure (a) depicts the blood glucose levels during these weeks. Figure (b) depicts the amount of insulin (bolus) injected during these weeks. Figure (c) depicts the amount of carbohydrate consumed in grams during these weeks. Table 3 shows the mean percentage change between these weeks. In all the figures, the asterisk shows the mean value and the red line depicts the median value for the week.

## The Third Case of Infection (flu)

1. Comparison of Blood Glucose levels during pre-infection week, infection week and post-infection week. As can be seen, the blood glucose is elevated during the infection week as compared to the pre and post-infection week.

1. Comparison of Insulin (bolus) intake during pre-infection week, infection week and post-infection week. As can be seen, the amount of insulin (bolus)intake is elevated during the infection week as compared to the pre and post-infection week.

1. Comparison of Carbohydrate (grams) intake during pre-infection week, infection week and post-infection week. As can be seen, the amount of Carbohydrate (grams) intake is significantly reduced during the infection week as compared to the pre and post-infection week.
2. **Table 4**: Mean Percentage Change between pre-infection week versus infection week and post-infection week and infection week. For further reference, see **Table 1** Above.

| **Parameters** | **Pre-infection Vs. Infection week** | **Post-infection week Vs. Infection week** |
| --- | --- | --- |
| **Blood Glucose** (mg/dl) | 7.26**%** | 7.41**%** |
| **Insulin (bolus)** | 56.87**%** | 37.98**%** |
| **Carbohydrate** (grams) | -18.63**%** | -26.04**%** |
| **Hourly** |  |  |
| **Blood Glucose** (mg/dl) | **4.56%** | 7.21**%** |
| **Insulin (bolus)** | **50.0%** | 61.29**%** |
| **Carbohydrate** (grams) | -15.95**%** | -10.99**%** |

**Figure 3**: Analysis of pre-infection week, infection week, and post-infection week based on the third patient year. Figure (a) depicts the blood glucose levels during these weeks. Figure (b) depicts the amount of insulin (bolus)injected during these weeks. Figure (c) depicts the amount of carbohydrate consumed in grams during these weeks. Table 4 shows the mean percentage change between these weeks. In all the figures, the asterisk shows the mean value and the red line depicts the median value for the week.

## The Fourth Case of Infection (flu)

1. Comparison of Blood Glucose levels during pre-infection week, infection week and post-infection week. As can be seen, the blood glucose is elevated during the infection week as compared to the pre and post-infection week.

1. Comparison of Insulin (bolus) intake during pre-infection week, infection week and post-infection week. As can be seen, the amount of insulin (bolus)intake is elevated during the infection week as compared to the pre and post-infection week.

1. Comparison of Carbohydrate (grams) intake during pre-infection week, infection week and post-infection week. As can be seen, the amount of Carbohydrate (grams) intake is significantly reduced during the infection week as compared to the pre and post-infection week.
2. **Table 5**: Mean Percentage Change between pre-infection week versus infection week and post-infection week and infection week. For further reference, see **Table 1** Above.

| **Parameters** | **Pre-infection Vs. Infection week** | **Post-infection week Vs. Infection week** |
| --- | --- | --- |
| **Blood Glucose** (mg/dl) | **2.28%** | 16.43**%** |
| **Insulin (bolus)** | **31.56%** | 9.73**%** |
| **Carbohydrate** (grams) | **-16.09%** | -35.34**%** |
| **Hourly** |  |  |
| **Blood Glucose** (mg/dl) | **4.03%** | 10.92**%** |
| **Insulin (bolus)** | **44.79%** | 16.81**%** |
| **Carbohydrate** (grams) | **-19.09%** | -30.68**%** |

**Figure 4**: Analysis of pre-infection week, infection week, and post-infection week based on the fourth patient year. Figure (a) depicts the blood glucose levels during these weeks. Figure (b) depicts the amount of insulin (bolus) injected during these weeks. Figure (c) depicts the amount of carbohydrate consumed in grams during these weeks. Table 5 shows the mean percentage change between these weeks. In all the figures, the asterisk shows the mean value and the red line depicts the median value for the week.

## The Fifth Case of Infection (flu)

1. Comparison of Blood Glucose levels during pre-infection week, infection week and post-infection week. As can be seen, the blood glucose is elevated during the infection week as compared to the pre and post-infection week.

1. Comparison of Insulin (bolus) intake during pre-infection week, infection week and post-infection week. As can be seen, the amount of insulin (bolus) intake is elevated during the infection week as compared to the pre and post-infection week.

1. Comparison of total Insulin (bolus and basal) intake during pre-infection week, infection week and post-infection week. As can be seen, the amount of total insulin intake is elevated during the infection week as compared to the pre and post-infection week.

1. Comparison of Insulin (basal) intake during pre-infection week, infection week and post-infection week. As can be seen, the amount of insulin (basal) intake is elevated during the infection week as compared to the pre and post-infection week.
2. **Table 6**: Mean Percentage Change between pre-infection week versus infection week and post-infection week and infection week. For further reference, see **Table 1** Above.

| **Parameters** | **Pre-infection Vs. Infection week** | **Post-infection week Vs. Infection week** |
| --- | --- | --- |
| **Blood Glucose** (mg/dl) | 3.45**%** | 13.84**%** |
| **Insulin (bolus)** | 23.08**%** | 21.01**%** |
| **Insulin (basal)** | 6.35**%** | 9.32**%** |
| **Total Insulin** | 16.97**%** | 16.68**%** |
| **Hourly** |  |  |
| **Blood Glucose** (mg/dl) | 7.346**%** | 17.99**%** |
| **Insulin (bolus)** | 29.42**%** | 21.38**%** |

**Figure 5**: Analysis of pre-infection week, infection week, and post-infection week based on the fifth patient year. Figure (a) depicts the blood glucose levels during these weeks. Figure (b) depicts the amount of insulin (bolus) injected during these weeks. Figure (c) depicts the amount of total insulin (bolus + basal) injected during these weeks. Figure (d) depicts the amount of insulin (basal) injected during these weeks. Table 6 shows the mean percentage change between these weeks. In all the figures, the asterisk shows the mean value and the red line depicts the median value for the week.

## **Kernel Density Estimation**

The kernel density was estimated relying on two procedures; by removing the infection period from the yearly data and computing the distribution and computing the kernel density for the whole year including the infection period. This is carried out so as to identify the effect of the infection period on the distribution of the data (please see the manuscript for further explanation).

## The First Case of infection (flu)

1. Kernel density estimation of daily total insulin (bolus) to carbohydrate ratio.

1. Kernel density estimation of hourly total insulin (bolus) to carbohydrate ratio.

**Figure 6**: Univariate kernel density estimation of a patient year using the daily insulin (bolus) to carbohydrate ratio. Figure (a) depicts the univariate kernel estimation of the daily total insulin (bolus) to carbohydrate ratio. Figure (b) depicts the univariate kernel estimation of the hourly total insulin (bolus) to carb ratio. As can be seen from the tail of the distribution, during normal days (the green shaded region) almost most of the yearly distribution of the patient insulin (bolus) to carbohydrate ratio lies within the values of 0.005 and 0.2. However, during infection incidence (the red shaded region) there is a clear deviation in the tail of the distribution, where the values reaches around 0.58.

1. Kernel density estimation of daily average blood glucose levels vs. total insulin (bolus) to carbohydrate ratio.

1. Kernel density estimation of hourly average blood glucose levels vs. total insulin (bolus) to carbohydrate ratio.

**Figure 7**: Bivariate Kernel Density estimation of a patient year using both the average BG levels and insulin (bolus) to carbohydrate ratio. Figure (a) depicts the bivariate kernel estimation of the daily average BG vs. total insulin (bolus) to carbohydrate ratio. Figure (b) depicts the bivariate kernel estimation of the hourly average BG vs. total insulin (bolus) to carbohydrate ratio. As can be seen from the bivariate distribution, during regular/normal days (the top light green figure), the distributions are concentrated around the high density regions. However, during infection incidence (the lower figure), there is a clear bump far from the high density regions.

## The Second Case of infection (flu)

1. Kernel density estimation of daily total insulin (bolus) to carbohydrate ratio.

1. Kernel density estimation of hourly total insulin to carbohydrate ratio.

**Figure 8**: Univariate Kernel Density estimation of a patient year using the daily insulin (bolus) to carbohydrate ratio. Figure (a) depicts the univariate kernel estimation of the daily total insulin (bolus) to carbohydrate ratio. Figure (b) depicts the univariate kernel estimation of the hourly total insulin (bolus) to carb ratio. As can be seen from the tail of the distribution, during regular/normal days (the green shaded region) almost most of the yearly distribution of the patient insulin to carbohydrate ratio lies within the values of 0.005 and 0.25. However, during infection incidence (the red shaded region) there is a clear deviation in the tail of the distribution, where the values reaches around 0.7.

1. Kernel density estimation of daily average blood glucose levels and total insulin (bolus) to carbohydrate ratio.

1. Kernel density estimation of hourly average blood glucose levels and total insulin (bolus) to carbohydrate ratio.

**Figure 9**: Bivariate Kernel Density estimation of a patient year using both the average blood glucose levels and insulin (bolus) to carbohydrate ratio. Figure (a) depicts the bivariate kernel estimation of the daily average BG vs. total insulin (bolus) to carbohydrate ratio. Figure (b) depicts the bivariate kernel estimation of the hourly average BG vs. total insulin (bolus) to carbohydrate ratio. As can be seen from the bivariate distribution, during regular/normal days (the top light green figure), the distributions are concentrated around the high density regions. However, during infection incidence (the lower figure), there is a clear bump far from the high density regions.

## The Third Case of infection (flu)

1. Kernel density estimation of daily total insulin (bolus) to carbohydrate ratio.

1. Kernel density estimation of hourly total insulin (bolus) to carbohydrate ratio.

**Figure 10**: Univariate Kernel Density estimation of a patient year using the daily insulin (bolus) to carbohydrate ratio. Figure (a) depicts the univariate kernel estimation of the daily total insulin (bolus) to carbohydrate ratio. Figure (b) depicts the univariate kernel estimation of the hourly total insulin (bolus) to carb ratio. As can be seen from the tail of the distribution, during regular/normal days (the green shaded region) almost most of the yearly distribution of the patient insulin (bolus) to carbohydrate ratio lies within the values of 0.005 and 0.26. However, during infection incidence (the red shaded region) there is a clear deviation in the tail of the distribution, where the values reaches around 0.5.

1. Kernel density estimation of daily average blood glucose levels and total insulin (bolus) to carbohydrate ratio.

1. Kernel density estimation of hourly average blood glucose levels and total insulin (bolus) to carbohydrate ratio.

**Figure 11**: Bivariate Kernel Density estimation of a patient year using both the average blood glucose levels and insulin (bolus) to carbohydrate ratio. Figure (a) depicts the bivariate kernel estimation of the daily average BG vs. total insulin (bolus) to carbohydrate ratio. Figure (b) depicts the bivariate kernel estimation of the hourly average BG vs. total insulin (bolus) to carbohydrate ratio. As can be seen from the bivariate distribution, during regular/normal days (the top light green figure), the distributions are concentrated around the high density regions. However, during infection incidence (the lower figure), there is a clear bump far from the high density regions.

## The Fourth Case of infection (flu)

1. Kernel density estimation of daily total insulin (bolus) to carbohydrate ratio.

1. Kernel density estimation of hourly total insulin (bolus) to carbohydrate ratio.

**Figure 12**: Univariate Kernel Density estimation of a patient year using the daily insulin (bolus) to carbohydrate ratio. Figure (a) depicts the univariate kernel estimation of the daily total insulin (bolus) to carbohydrate ratio. Figure (b) depicts the univariate kernel estimation of the hourly total insulin (bolus) to carb ratio. As can be seen from the tail of the distribution, during regular/normal days (the green shaded region) almost most of the yearly distribution of the patient insulin to carbohydrate ratio lies within the values of 0.005 and 0.2. However, during infection incidence (the red shaded region) there is a clear deviation in the tail of the distribution, where the values reaches around 0.45.

1. Kernel density estimation of daily average blood glucose levels and total insulin (bolus) to carbohydrate ratio.

1. Kernel density estimation of hourly average blood glucose levels and total insulin (bolus) to carbohydrate ratio.

**Figure 13**: Bivariate Kernel Density estimation of a patient year using both the average blood glucose levels and insulin (bolus) to carbohydrate ratio. Figure (a) depicts the bivariate kernel estimation of the daily average BG vs. total insulin (bolus) to carbohydrate ratio. Figure (b) depicts the bivariate kernel estimation of the hourly average BG vs. total insulin (bolus) to carbohydrate ratio. As can be seen from the bivariate distribution, during regular/normal days (the top light green figure), the distributions are concentrated around the high density regions. However, during infection incidence (the lower figure), there is a clear bump far from the high density regions.
