# Supplementary material for: Toward Detecting Infection Incidence in People With Type 1 Diabetes Using Self-Recorded Data (Part 1): A Novel Framework for a Personalized Digital Infectious Disease Detection System
Source: J Med Internet Res. 2020 Aug 12;22(8):e18911. doi: 10.2196/18911 (PMC7450374; doi:10.2196/18911)
Supplement: Multimedia Appendix 2 [file jmir_v22i8e18911_app2.docx]

# **Appendix 2: Analytical plot of the Normal/Regular Patient Years**

The normal/regular patient year depicts the absence of significant infection incidences on the individual patient. These data are used to validate the change associated with presence of infections in an individual. The data were analyzed after computing the daily or hourly average BG, total insulin and carbohydrate consumption and smoothing with a 2-days window size moving average filter. The data was filtered to remove short term noise. The analytical plot as shown in the figure below demonstrated that under normal conditions the insulin to carbohydrate ratio remains between 0.05 and 0.2 in all the normal patient years.

## The First Patient Year

1. Daily average BG levels, total insulin (bolus), total carbohydrate, and insulin to carbohydrate ratio.

1. Hourly average BG levels, total insulin (bolus), total carbohydrate, and insulin to carbohydrate ratio.

**Figure 1**: The first patient year, where there is no incidence of acute infections. Figure (a) depicts the daily variation of BG, total insulin (bolus), carbohydrate, and insulin to carbohydrate ratio. Figure (b) depicts variation of the same variable during each hours of the day. The operating point of the patient’s insulin to carbohydrate ratio through these normal days is between 0.05 to 0.2.

## The Second Patient Year

1. Daily average BG levels, total insulin (bolus), total carbohydrate, and insulin to carbohydrate ratio.

1. Hourly average BG levels, total insulin (bolus), total carbohydrate, and insulin to carbohydrate ratio.

**Figure 2**: The second patient year, where there is no incidence of acute infections. Figure (a) depicts the daily variation of BG, total insulin (bolus), carbohydrate, and insulin to carbohydrate ratio. Figure (b) depicts variation of the same variable during each hours of the day. The operating point of the patient’s insulin to carbohydrate ratio through these normal days is between 0.05 to 0.2.

## The Third Patient Year

1. Daily average BG levels, total insulin (bolus), total carbohydrate, and insulin to carbohydrate ratio.

1. Hourly average BG levels, total insulin (bolus), total carbohydrate, and insulin to carbohydrate ratio.

**Figure 3**: The third patient year, where there is no incidence of acute infections. Figure (a) depicts the daily variation of BG, total insulin (bolus), carbohydrate, and insulin to carbohydrate ratio. Figure (b) depicts variation of the same variable during each hours of the day. The operating point of the patient’s insulin to carbohydrate ratio through these normal days is between 0.05 to 0.2.

## The Fourth Patient Year

1. Daily average BG levels, total insulin (bolus), total carbohydrate, and insulin to carbohydrate ratio.

1. Hourly average BG levels, total insulin (bolus), total carbohydrate, and insulin to carbohydrate ratio.

**Figure 4**: The fourth patient year, where there is no incidence of acute infections. Figure (a) depicts the daily variation of BG, total insulin (bolus), carbohydrate, and insulin to carbohydrate ratio. Figure (b) depicts variation of the same variable during each hours of the day. The operating point of the patient’s insulin to carbohydrate ratio through these normal days is between 0.05 to 0.21.

## The Fifth Patient Year

1. Daily average BG levels, total insulin (bolus), total carbohydrate, and insulin to carbohydrate ratio.

1. Hourly average BG levels, total insulin (bolus), total carbohydrate, and insulin to carbohydrate ratio.

**Figure 5**: The fifth patient year, where there is no incidence of acute infections. Figure (a) depicts the daily variation of BG, total insulin (bolus), carbohydrate, and insulin to carbohydrate ratio. Figure (b) depicts variation of the same variable during each hours of the day. The operating point of the patient’s insulin to carbohydrate ratio through these normal days is between 0.05 to 0.22.
