# Supplementary material for: Toward Detecting Infection Incidence in People With Type 1 Diabetes Using Self-Recorded Data (Part 1): A Novel Framework for a Personalized Digital Infectious Disease Detection System
Source: J Med Internet Res. 2020 Aug 12;22(8):e18911. doi: 10.2196/18911 (PMC7450374; doi:10.2196/18911)
Supplement: Multimedia Appendix 3 [file jmir_v22i8e18911_app3.docx]

# **Appendix 3: Analytical plot of the Patient Years with acute infection**

The patient year with acute infection depicts a patient year containing at least with one or more infection incidences. These data are used to compare and evaluate the effect of infection incidences on the key parameters of the BG dynamics on an individual basis including BG levels, insulin intake, carbohydrate consumption, and ratio of insulin to carbohydrate. The data were analyzed after computing the daily or hourly average BG, total insulin and carbohydrate consumption and smoothing with a 2-days window size moving average filter. The data was filtered to remove short term noise. The analytical plot as shown in the figure below demonstrated that during infection incidences the key parameters of the BG dynamics are highly affected. The patient experiences elevated BG levels while the insulin to carbohydrate ratio is dramatically shifted to a higher value depicting higher insulin intake with low carbohydrate ingestions. During normal conditions the insulin to carbohydrate ratio remains between 0.05 and 0.2.

## The first case of infection (Flu)

1. Daily average BG levels, total insulin (bolus), total carbohydrate, and insulin to carbohydrate ratio based on a window size of two days.

1. Hourly average BG levels, total insulin (bolus), total carbohydrate, and insulin to carbohydrate ratio based on a window size of 48 hours.

**Figure 1**: The sixth patient year, where the patient was infected with influenza (flu) starting from the first week of December. Figure (a) depicts the daily variation of BG, total insulin (bolus), carbohydrate, and insulin to carbohydrate ratio. Figure (b) depicts variation of the same variable during each hours of the day. The operating point of the patient’s insulin to carbohydrate ratio had dramatically shifted and raised above the regular/normal days and reach a top around 0.5 upon mid infection week.

## The second case of infection (Flu)

1. Daily average BG levels, total insulin (bolus), total carbohydrate, and insulin to carbohydrate ratio based on a window size of two days.

1. Hourly average BG levels, total insulin (bolus), total carbohydrate, and insulin to carbohydrate ratio based on a window size of 48 hours.

**Figure 2**: The seventh patient year, where the patient was infected with influenza (flu) starting from the first week of April. Figure (a) depicts the daily variation of BG, total insulin (bolus), carbohydrate, and insulin to carbohydrate ratio. Figure (b) depicts variation of the same variable during each hours of the day. The operating point of the patient’s insulin to carbohydrate ratio had dramatically shifted and raised above the regular/normal days and reach a top around 0.45 upon mid infection week.

## The third case of infection (Flu)

1. Daily average BG levels, total insulin (bolus), total carbohydrate, and insulin to carbohydrate ratio based on a window size of two days.

1. Hourly average BG levels, total insulin (bolus), total carbohydrate, and insulin to carbohydrate ratio based on a window size of 48 hours.

**Figure 3**: The eighth patient year, where the patient was infected with influenza (flu) starting from the last week of November. Figure (a) depicts the daily variation of BG, total insulin (bolus), carbohydrate, and insulin to carbohydrate ratio. Figure (b) depicts variation of the same variable during each hours of the day. The operating point of the patient’s insulin to carbohydrate ratio had dramatically shifted and raised above the normal days and topped around 0.4 upon mid infection week.

## The fourth case of infection (Flu)

1. Daily average BG levels, total insulin (bolus), total carbohydrate, and ratio of insulin to carbohydrate based on a window size of two days.

1. Hourly average BG levels, total insulin (bolus), total carbohydrate, and insulin to carbohydrate ratio based on a window size of 48 hours.

**Figure 4**: The ninth patient year, where the patient was infected with three series of acute infection (mild common cold without fever starting on the first week of august and light common cold without fever starting of mid-February, influenza (flu) starting from mid-August). Figure (a) depicts the daily variation of BG, total insulin (bolus), carbohydrate, and insulin to carbohydrate ratio. Figure (b) depicts variation of the same variable during each hours of the day. The operating point of the patient’s insulin to carbohydrate ratio had dramatically shifted and raised above the normal days and reach a top around 0.28 upon mid infection week. A light common cold without fever seems to not significantly affect the operating point.

## The fifth case of infection (Flu)

1. Daily average BG levels, total insulin including both bolus and basal insulin, daily average rate of change of CGM and absolute value of rate of change of CGM (computed based on CGM direction from the pump information), percentage of basal and bolus per total insulin units based on a window size of two days.

1. Hourly average BG levels, total insulin (bolus), hourly average rate of change of CGM (computed based on CGM direction from the pump information) based on a window size of 48 hours.

**Figure 5**: The tenth patient year, where the patient was infected with influenza (flu) starting from mid-January. The first Figure (a) depicts the daily variation and Figure (b) depicts variation during each hours of the day. As can be seen, there is clear and dramatic rise in insulin amount, while the BG levels remain elevated due to the ongoing infection incidence.
